# DST-3, a Novel Modified Cryptotanshinone, Protects Against Pulmonary Fibrosis via Inhibiting STAT3/Smad Signaling Pathway and Improves Bioavailability

**DOI:** 10.3390/pharmaceutics17101307

**Published:** 2025-10-08

**Authors:** Ruoqing Guan, Xiangjun He, Yuxing Dai, Guangye Huang, Zhaoyun Xue, Jianwen Chen, Peiqing Liu

**Affiliations:** 1 National and Local United Engineering Lab of Druggability and New Drugs Evaluation, School of Pharmaceutical Sciences, Sun Yat-sen University, Guangzhou 510006, China; guanrq@mail2.sysu.edu.cn (R.G.); hexj55@mail3.sysu.edu.cn (X.H.); daiyx5@mail2.sysu.edu.cn (Y.D.); huanggy29@mail.sysu.edu.cn (G.H.); xuezhy3@mail.sysu.edu.cn (Z.X.); 2 Guangdong Province Engineering Laboratory for Druggability and New Drug Evaluation, School of Pharmaceutical Sciences, Sun Yat-sen University, Guangzhou 510006, China

**Keywords:** DST-3, pulmonary fibrosis, pharmacokinetics, HPLC, tandem mass spectrometry, cryptotanshinone, STAT3 transcription factor

## Abstract

**Background/Objectives**: Idiopathic pulmonary fibrosis (IPF) is a fatal lung disease characterized by progressive loss of lung function and poor prognosis. Cryptotanshinone (CTS), a small-molecule compound extracted from Salvia miltiorrhiza, possesses diverse pharmacological activities but suffers from poor oral bioavailability, which restricts its clinical development, particularly in pulmonary fibrosis. DST-3, a newly synthesized derivative of CTS, was designed to overcome these limitations. **Methods**: The antifibrotic effects of DST-3 were investigated in a bleomycin-induced pulmonary fibrosis model in C57BL/6 mice through lung function assessment, histopathological evaluation, hydroxyproline quantification, and cytokine profiling. In vitro, TGF-β1-stimulated MRC5 fibroblasts were employed to explore the mechanism of action, focusing on STAT3/Smad signaling via Western blotting and molecular binding assays. Furthermore, a validated HPLC–MS/MS method was developed for DST-3, and its pharmacokinetic profile was characterized in Sprague–Dawley rats and compared with that of CTS. **Results**: DST-3 markedly attenuated pulmonary fibrosis in vivo, as evidenced by improved lung function, reduced collagen deposition, and decreased proinflammatory cytokine levels. In vitro, DST-3 inhibited TGF-β1-induced fibroblast activation by directly binding to STAT3 and suppressing STAT3/Smad signaling. Pharmacokinetic analysis demonstrated that, compared with CTS, DST-3 exhibited more rapid absorption, a higher peak plasma concentration, a greater area under the curve (AUC), improved hepatic metabolic stability, and enhanced lung tissue exposure. **Conclusions**: Our study demonstrates that DST-3 exerts potent antifibrotic effects in vivo and in vitro, primarily through STAT3 pathway inhibition. Its improved pharmacokinetic characteristics further support its potential as a promising candidate for the treatment of pulmonary fibrosis.

## 1. Introduction

Idiopathic pulmonary fibrosis (IPF) is the most prevalent and devastating form of interstitial lung disease, characterized by progressive scarring of lung tissue and limited therapeutic options [1]. It predominantly affects older males, with incidence rising markedly with advancing age [2]. Unfortunately, the prognosis remains poor, with a median survival of merely 2.5 to 3.5 years following diagnosis [3,4]. Clinically, IPF is characterized by progressive decline in lung function, culminating in respiratory failure and death. However, no curative therapies are available by now. Currently, the two FDA-approved drugs, pirfenidone and nintedanib, can moderately slow disease progression, yet their clinical utility is limited by substantial side effects [5,6]. The pathogenesis of pulmonary fibrosis remains incompletely understood, although emerging evidence has implicated genetic susceptibility [7], cigarette smoking [8], environmental pollutants [9], and viral infections [10]—including Epstein–Barr virus, cytomegalovirus, and SARS-CoV-2—as potential contributing factors [11,12,13].

Pulmonary fibrosis is characterized pathologically by persistent alveolar epithelial injury, chronic inflammation, fibroblast proliferation and activation, and excessive extracellular matrix (ECM) deposition, culminating in progressive parenchymal scarring and irreversible loss of lung function [14,15,16,17]. Increasing evidence suggests that multiple signaling pathways are involved in this dysregulated repair process. Notably, the JAK/STAT3 pathway is pivotal in driving fibroblast-to-myofibroblast differentiation, inflammatory cytokine production, and extracellular matrix (ECM) accumulation [18,19,20]. Aberrant STAT3 activation is consistently detected in fibrotic lung tissues and closely associates with disease severity [21]. Thus, targeting STAT3 or its upstream JAK kinases represents a promising therapeutic avenue to suppress fibrotic signaling and potentially reverse tissue remodeling in IPF.

Cryptotanshinone (CTS) is a fat-soluble diterpenoid quinone compound isolated from the roots of Salvia miltiorrhiza [22,23]. It possesses several favorable pharmaceutical properties, including low toxicity, a relatively small molecular weight, and diverse pharmacological activities, such as cardiovascular and cerebrovascular protection, anti-inflammatory, antioxidant, anti-fibrotic, and anti-cancer effects [24,25,26,27,28]. Pharmacokinetic studies have shown that CTS is widely distributed in various tissues, especially in the liver and lungs [29]. In our previous work, we also observed increased plasma concentrations and enhanced lung distribution of CTS in Sprague–Dawley (SD) rats with bleomycin (BLM)-induced pulmonary fibrosis compared to healthy controls [30]. Despite its promising pharmacological profile, the clinical application of CTS remains limited due to its photosensitivity and suboptimal oral bioavailability (typically below 10%), which primarily result from its low water solubility and poor dissolution behavior [31,32]. Although various delivery strategies—including cyclodextrin inclusion complexes and soluble microparticle systems—have been explored to enhance the solubility and dissolution rate of CTS [33,34,35], their actual impact on improving oral bioavailability remains unclear due to limited in vivo data. To overcome these limitations and enhance the pharmaceutical properties of CTS, structural modifications are required to improve its oral bioavailability.

In this study, we synthesized a CTS derivative, termed N-(pyridin-2-yl)-(1R)-1-(4-chlorobenzyl)-6-hydroxy-7-methyl-3,4-dihydrophenanthrene-2,4-dicarboxamide (DST-3), to enhance its drug accessibility, and systematically investigated its therapeutic effects and underlying regulatory mechanisms in pulmonary fibrosis. In addition, we also referred to the LC-MS/MS method established by Song [36] to study the pharmacokinetics of CTS in SD rats and constructed and verified a brand new HPLC-MS/MS method. Moreover, the method was demonstrated to be sensitive, efficient, and stable for PK studies of DST-3 in normal rats. These efforts were designed to provide a comprehensive evaluation of DST-3’s pharmacological properties and bioavailability relative to CTS.

## 2. Materials and Methods

### 2.1. Chemical Agents and Antibodies

DST-3 (purity >95%) was obtained from the School of Pharmacy, Sun Yat-sen University (Guangzhou, China). It was selected from a series of CTS derivatives based on preliminary cytotoxicity evaluation, exhibiting the lowest cytotoxicity (see Supplementary Material S1). The synthesis method, route, and structure of DST-3 are detailed in Supplementary Material S1. Drugs and chemicals were sourced as follows CTS and HPLC-grade CTS standard (YuanYe, Shanghai, China); Loratadine (LTD) standard (China Pharmaceutical and Biological Products Inspection Institute, Beijing, China); Methanol (HPLC-grade, Amethyst, Shanghai, China); Ethyl acetate (HPLC-grade, Kermel, Tianjin, China); Formic acid (HPLC-grade, Aladdin, Shanghai, China); Ultra-pure water, made by the laboratory; Bleomycin (Macklin, Shanghai, China); Hydroxyproline test kit (Jiancheng Bioengineering Institute, Nanjing, China); Sodium carboxymethyl cellulose (Tianjin Zhiyuan Chemical Reagent Co., Ltd., Tianjin, China); Normal saline (Jiangxi Kelun Pharmaceutical Co., Ltd., Jiangxi, China); Sodium pentobarbital (Beijing Huanye World Chemical Co., Ltd., Beijing, China); 4% paraformaldehyde (Servicebio, Wuhan, China); Human TGF-β1 recombinant protein (PeproTech, Cranbury, NJ, USA). All of primary antibodies or secondary antibodies are provided in Supplementary Material S2.

### 2.2. Animals and Experiment

The study was conducted in accordance with the recommendations of the Guidelines for the Care and Use of Laboratory Animals (NIH Publication No. 85-23, revised 1996). Male SD rats (220.0~260.0 g, SPF grade) and male C57BL/6 mice (18.0~22.0 g, SPF grade, 8 weeks of age) were provided by the Animal Experimental Center, East Campus of Sun Yat-sen University (approved by the Ethics Committee of Sun Yat-sen University, approval No: SYSU-IACUC-2022-000403; approval date: 14 February 2022) and raised in an SPF environment. Animals were kept under quarantine for an observation period of 5 days. Animals were in good health and exhibited no anomalous behavior or activities when their appearance, physical characteristics, behavioral activity, weight, and diet were evaluated during this time.

#### 2.2.1. Pharmacodynamics Study

Sixty male Sprague-Dawley (SD) rats were individually weighed one day prior to administration and randomly assigned to six groups (*n* = 10 per group) using a stratified randomization method to ensure balanced average body weights across groups (weight range: 220–260 g; inter-group mean weight difference < 5 g). The vehicle used for intravenous administration of both CTS and DST-3 was 0.5% sodium carboxymethyl cellulose (CMC-Na) solution. A–F:

(A) Normal saline + CMC–Na;
(B) BLM + 0.5% CMC–Na;
(C) BLM + DST-3 15 mg/kg;
(D) BLM + DST-3 30 mg/kg;
(E) BLM + DST-3 60 mg/kg;
(F) BLM + CTS 30 mg/kg.

Pulmonary fibrosis was induced in groups B–F by tracheal infusion of bleomycin 2 mg/kg, and in group A by normal saline. To create a suspension for oral administration, all DST-3 or CTS were dissolved in 0.5% CMC-Na. After modeling for 21 days, the C–F group began receiving oral administration of various doses of DST-3 or CTS (twice a day, 12 h—interval, morning at 9:00 a.m. and evening at 9:00 p.m.), whereas the A and B groups received only 0.5% CMC-Na.

#### 2.2.2. Pharmacokinetic Study

A total of 16 male SD rats, according to weight, were randomly divided into four groups (*n* = 4), a~d, as described in pharmacodynamics study:

(A) DST-3 intravenous injection of 6 mg/kg;
(B) DST-3 oral gavage of 60 mg/kg;
(C) CTS intravenous injection of 6 mg/kg;
(D) CTS oral gavage of 60 mg/kg.

Blood collection was performed in all rats at 0.25 h, 0.5 h, 1 h, 2 h, 3 h, 4 h, 6 h, 8 h, 12 h, and 24 h after administration.

#### 2.2.3. Tissue Distribution Study

A total of 32 male C57BL/6 mice were randomly divided into 2 groups (e and f, *n* = 16) as described in pharmacodynamics study, and each group was further divided into 4 subgroups according to body weight (*n* = 4):

(E) DST-3 oral gavage of 60 mg/kg (0.5 h group, 3 h group, 10 h group, 24 h group).
(F) CTS oral gavage of 60 mg/kg (0.5 h group, 3 h group, 10 h group, 24 h group).

The heart, liver, spleen, lung, kidney, and brain tissues were, respectively, obtained at 0.5 h, 3 h, 10 h, and 24 h after administration.

### 2.3. Pulmonary Function Assay

Animal lung function test system (WBP-4A, EMKA Technologies, Paris, France) was used to collect lung function indexes of mice in each group. In a silent setting with constant air flow, mice were inserted into a whole-body plethysmography system. After the mouse rested, data were gathered continuously for more than 5 min, and the mean value within 5 min was taken for statistical analysis.

### 2.4. Morphological and Histology Analysis

On the 22nd day, mice were sacrificed in all groups, and their whole lungs were swiftly removed. The entire left lung leaf was preserved by spending the night in 4% paraformaldehyde, then sliced into 5 μm sections, and embedded in paraffin blocks. To evaluate the histopathological changes in the lungs, sections were stained with hematoxylin-eosin (HE) and Masson, and images were taken under an optical microscope (EVOS FL Auto Cell Imaging System, Thermo Fisher Scientific, Waltham, MA, USA).

### 2.5. Measurement of Hydroxyproline (HYP) Assay

HYP was specifically detected in collagen as a posttranslational product of proline hydroxylation. The content of HYP in tissues not only reflects the level of collagen but also serves as a marker of fibrosis [37]. In this study, the content of HYP in lung tissue was determined by alkaline hydrolyzation [38]. Fresh right lung tissue (wet weight 80–100 mg) was accurately weighed and hydrolyzed in test tubes containing 1 mL of the cracking solution (2 mol/L NaOH, used for alkaline hydrolysis). After the tube was cooled, the pH of the cracking solution was adjusted to 6.0~6.8, and 10 mL of double-steamed water was added. The supernatant was taken after centrifugation, and t and processed following the instructions of the HYP test kit (#A030-2, Nanjing Jianchen Institute of Biological Engineering, Nanjing, China). Each sample was measured at 550 nm to detect the absorbance, and the HYP content was calculated according to the following formula:

$$\mathrm{Hydroxproline}\;\mathrm{content}\;(\mu g/\mathrm{mg})\;=\;\frac{\mathrm{Measured}\;\mathrm{OD}\;\mathrm{value}\;-\mathrm{Blank}\;\mathrm{OD}\;\mathrm{value}}{\mathrm{Standard}\;\mathrm{OD}\;\mathrm{value}\;-\mathrm{Blank}\;\mathrm{OD}\;\mathrm{value}}\times \;\mathrm{Stand}\;\mathrm{content}\;\times \frac{\mathrm{Lysate}\;\mathrm{total}\;\mathrm{volume}\;(\mathrm{mL})}{\mathrm{Organization}\;\mathrm{wet}\;\mathrm{weight}\;(\mathrm{mg})}$$

### 2.6. Enzyme-Linked Immunosorbent Assay (ELISA)

Bronchoalveolar lavage fluid (BALF) was collected by injecting 1 mL of cold phosphate-buffered saline (PBS) into the trachea three times, and the recovery rate was over 80%. The secretion levels of TNF-α (#70-EK282/4-96) and IL-6 (#70-EK206/3-96) in the cell-free supernatant of BALF samples were evaluated by ELISA kits (MultiSciences, Hangzhou, China).

### 2.7. Cell Culture

Medical Research Council cell strain-5 (MRC5, ATCC, Manassas, VA, USA, Cat# CCL-171) was supplemented with 10% fetal bovine serum (GIBCO, Invitrogen, Carlsbad, CA, USA) in low-sugar DMEM medium (GIBCO, Invitrogen, Carlsbad, CA, USA) in an incubator containing 5% CO_2_ atmosphere at 37 °C.

### 2.8. Cell Viability Assay

The cells were seeded in 96-well plates for 24 h and treated with DST-3 (1.25, 2.5, 5, 10, 20 μM) at different concentrations for 48 h. The CCK8 kit (Beyotime, Shanghai, China) was used to test the cytotoxic effect of DST-3 on MRC5 cells. Medium solution of 10% CCK8 was added to each well and incubated at 37 °C for 2 h. Absorbance was measured at 450 nm (BioTek, Elx800, Winooski, VT, USA).

### 2.9. Immunofluorescence Assay

MRC5 cells were fixed in 4% paraformaldehyde for 0.5 h, then permeated with 1% Triton X-100 (Sangon Biotech, Shanghai, China) for 10 min, and blocked with goat serum (BOSTER, Wuhan, China) for 1 h. The cells were then incubated with α-SMA or STAT3 (1:100 diluted) primary antibody overnight at 4 °C and secondary antibody at room temperature for 1 h. The nuclei were stained at room temperature with Hoechst 33,342 (1:100 diluted) for 10 min. Fluorescence images were captured using a laser scanning ultra-high resolution microscope (FV3000, Olympus Corporation, Tokyo, Japan).

### 2.10. Surface Plasmon Resonance (SPR)

The binding affinity between DST-3 and STAT3 was analyzed using Biacore 8K and Biacore Insight Evaluation software (version 6.0). Purified STAT3 protein (0.17 mg/mL) was dissolved in PBS and immobilized on CM5 chip (GE Healthcare, Chicago, IL, USA). DST-3 dissolved in the running buffer (filtered 1 × PBS containing 5% DMSO) flows through the chip to generate a response signal. Dynamics and affinity were calculated by Biacore Insight Evolution software, and the results were determined as binding affinity (Kd).

### 2.11. Molecular Docking

Molecular docking was performed using the Molecular Operating Environment (MOE, version 2022.02; Chemical Computing Group, Montreal, QC, Canada). The crystal structure of STAT3 (PDB ID: 6NUQ) was obtained from the Protein Data Bank (http://www.pdb.org), and all water molecules were removed during preprocessing. The three-dimensional structure of DST-3 was initially constructed using ChemDraw 3D (version 21.0.0) and further optimized by energy minimization to obtain a stable conformation suitable for docking.

The processed STAT3 structure was loaded into MOE, with the docking site defined in “All Atoms” mode to enable comprehensive atomic-level analysis of the binding pocket. DST-3 was subsequently introduced for docking. During the docking process, STAT3 was treated as a rigid receptor, whereas DST-3 was treated as a flexible ligand. Docking calculations and scoring were performed using MOE’s default parameters, and binding poses along with interaction energies were analyzed directly within the software.

### 2.12. Cellular Thermal Shift Assay

When the fusion density of MRC5s reached 70–80%, DMSO or DST-3 was added to the control group and the drug administration group for 1 h, respectively, and digestion centrifugation was performed. PBS with protease and phosphatase inhibitors (Bimake, Houston, TX, USA) was divided into EP tubes. The EP tubes were heated at the specified temperature for 2 min and then placed at room temperature for 3 min. The cell suspensions were rapidly frozen in liquid nitrogen and then thawed at room temperature. This freeze–thaw cycle was repeated three times to ensure complete cell lysis. Finally, centrifugation was performed at 4 °C at 12,000× *g* for 20 min, and the supernatant was collected. Cell samples were boiled for 5 min at 100 °C with loading buffer, and Western blot analysis was performed.

### 2.13. Western Blotting (WB)

MRC5 cells or lung tissues were washed with cold PBS and then lysed on ice using RIPA buffer (Beyotime Biotechnology, Shanghai, China) supplemented with protease and phosphatase inhibitors (Bimake, Boston, USA) for 0.5 h and centrifuged at 4 °C at 12,000× *g* for 15 min. Diocinonic acid (BCA protein Assay Kit, Pierce, Waltham, MA, USA) was used to determine protein concentration. Identical amounts of protein (30 μg for cell samples and 50 μg for tissue samples) were loaded into SDS-PAGE gels and transferred to polyvinylidene difluoride (PVDF) membrane (Millipore, Billerica, MA, USA). At room temperature, the membrane was sealed in a Tris-buffered brine Tween-20 buffer containing 5% (by weight/volume) skimmed milk powder for 1.5 h. The membrane was incubated with primary antibody (Table S2-1, Supplement Material S2) at 4 °C for 24 h and then incubated with secondary antibody at room temperature for 1.5 h. The substrate was washed three times with Tris-Buffered Saline with Tween 20 (TBST), and after 10 min each time, color bands were developed using an enhanced chemiluminescent reagent (High-sig ECL Western blot reagent, Tanon, Shanghai, China). Band strength was quantified by ImageJ software (version 1.53t, Bio-Rad, Rockford, IL, USA).

### 2.14. Sample Processing

The blank plasma for method validation was rapidly extracted from the main abdominal vein of SD rats. The sample for the pharmacokinetic study was obtained by taking 250 μL of blood from the orbit of SD rats. The blood was placed in a heparinized collection vessel, centrifuged at 3000 rpm for 10 min, and the upper plasma layer was taken.

According to the subgroups, the blood was flushed out through cardiac perfusion, and the heart, liver, spleen, lung, kidney, and brain tissues were extracted. Tissues were precisely weighed, and normal saline was added at the rate of 0.5 g/mL to prepare the homogenate. The supernatant homogenate was centrifuged at 12,000× *g* for 15 min.

A 100 μL sample was precisely measured, and 10 μL of loratadine working solution and 500 μL of ethyl acetate were added for liquid–liquid-extraction. The mixture was shaken for 1 min, centrifuged for 4 min at 12,000× *g*, and 400 μL of the upper organic phase was vacuum dried for 2 h. Then 100 μL of mobile phase (methanol-1% formic acid water (90:10, *v*/*v*)) was added, dissolved, vortexed for 1 min, and centrifuged at low temperature for 3 min at 12,000× *g*. The supernatant (80 μL) was placed in a sample bottle for HPLC-MS/MS analysis.

### 2.15. HPLC-MS/MS Conditions

#### 2.15.1. Mass Spectrum Condition

The DST-3 ion source used an electrospray ionization source (ESI) and positive ion scanning mode. The scanning mode was selected reaction monitor (SRM). Spray voltage: 3000 V; Sheath gas: 50 psi; Auxiliary gas: 10 psi; Capillary temperature: 350 °C; Peak width of color filter: 20.0 s; Impact gas pressure: 1.9 mtorr; Scanning width: 0.7 *m/z*; Scanning time: 0.1 s. The energy of ion reaction pairs and collisions and the parameters of mass-spectrometry are shown in Table 1 and Table 2.

#### 2.15.2. Chromatographic Condition

HyPURITY C18 (i.d. 2.1 × 50 mm, 3 mm, Thermo Scientific, US) column and EasyGuard C18 precolumn (10 × 4.0 mm, particle size: 5 mm) were used for analysis. Column temperature: 30 °C; mobile phase: methanol-1% formic acid water (90:10, *v*/*v*); sample size: 5 mL; sampling time: 6 min; flow rate: 0.2 mL/min.

The mass spectrometry and chromatographic conditions used for all CTS are referenced from the developed HPLC-MS/MS quantitative method [30].

### 2.16. Preparation of Stock Solution, Working Solution, and Quality Control Sample

DST-3, CTS, and LTD reference materials were accurately weighed and dissolved in methanol to prepare 1 mg/mL DST-3, CTS, and LTD reserve solutions. To generate a calibration curve, the working fluid was plotted into blank rat plasma or tissues, and the following concentrations were obtained:

(1) The DST-3 reserve solution was gradually diluted with 50% methanol to obtain a DST-3 standard curve working solution with a concentration of 20–2000 ng/mL. Quality control (QC) samples were prepared at low, medium, and high concentrations of 20, 200, and 2000 ng/mL, respectively.
(2) The CTS reserve solution was gradually diluted with 50% methanol to obtain a CTS standard curve working solution with a concentration of 20–2000 ng/mL. QC samples were prepared at low, medium, and high concentrations of 20, 200, and 2000 ng/mL, respectively.
(3) The LTD reserve liquid was diluted step by step with 50% methanol to obtain 200 ng/mL LTD internal standard working liquid.

All samples were stored at 4 °C prior to HPLC-MS/MS analysis.

### 2.17. Method Validation

The established HPLC-MS/MS method was verified in terms of specificity, linearity, lower limit of quantitation (LLOQ), precision, accuracy, sample stability, matrix effect, and recovery.

Specificity was assessed by comparing chromatograms of drug-free blank samples, low-concentration DST-3 QC samples, and oral 60 mg/kg DST-3 treated biological samples. The weighted least squares regression method was used for linear regression analysis. The X-coordinate is the drug concentration of DST-3 (ng/mL), and the Y-coordinate is the ratio of DST-3 to the chromatographic peak area. High, medium, and low QC samples were used for intraday and interday accuracy and precision assessment at 5 repetitions within 1 day and 3 consecutive days, respectively. Samples stored at room temperature for 48 h or −80 °C for one month were studied for stability. Matrix effects were evaluated using six batches of high, medium, and low QC samples with a normalized coefficient of variation in matrix factors not greater than 15%. More detailed methodological validation is presented in Supplementary Material S3.

### 2.18. Metabolic Stability of Liver Microsome Study

The mixed reaction system of 750 μL was prepared by adding 10 mg/mL DST-3 or CTS to the buffer containing 100 μL liver particle diluent (100 mM potassium phosphate buffer, pH = 7.4, supplemented with 3.3 mM MgCl_2_). Then the newly configured NADPH Regenerating System (NRS) excitation reaction system was added at 0 min, 30 min, 50 min, and 60 min, respectively. The reaction was immediately terminated with glacial acetic acid. After extraction by ethyl acetate, the established HPLC-MS/MS quantitative analysis method was used for sampling detection.

### 2.19. Pharmacokinetic Data and Statistical Analysis

According to the non-compartmental model, the pharmacokinetic software DAS 2.0 was used to calculate the main pharmacokinetic parameters: maximum plasma concentration (C_max_), elimination half-life (t_1/2_), time to reach maximum plasma concentration (T_max_), mean residence time (MRT_0–t_), area under the plasma concentration curve from 0 to the last measured concentration (AUC_0−t_), AUC from 0 to infinity (AUC_0–∞_), apparent clearance rate (CL/F) and apparent distribution volume (V/F).

The experimental data were statistically processed by biostatistical software (GraphPad Prism 9.0, San Diego, CA, USA) and expressed as mean ± SD. Unpaired Student’s test (*t*-test) was used to compare differences between two groups. One-way analysis of variance (ANOVA) with Bonferroni post hoc test was used to compare differences among various groups.

## 3. Results

### 3.1. DST-3 Prevented BLM-Induced Pulmonary Fibrosis in C57BL/6 Mice

**Figure 1:**
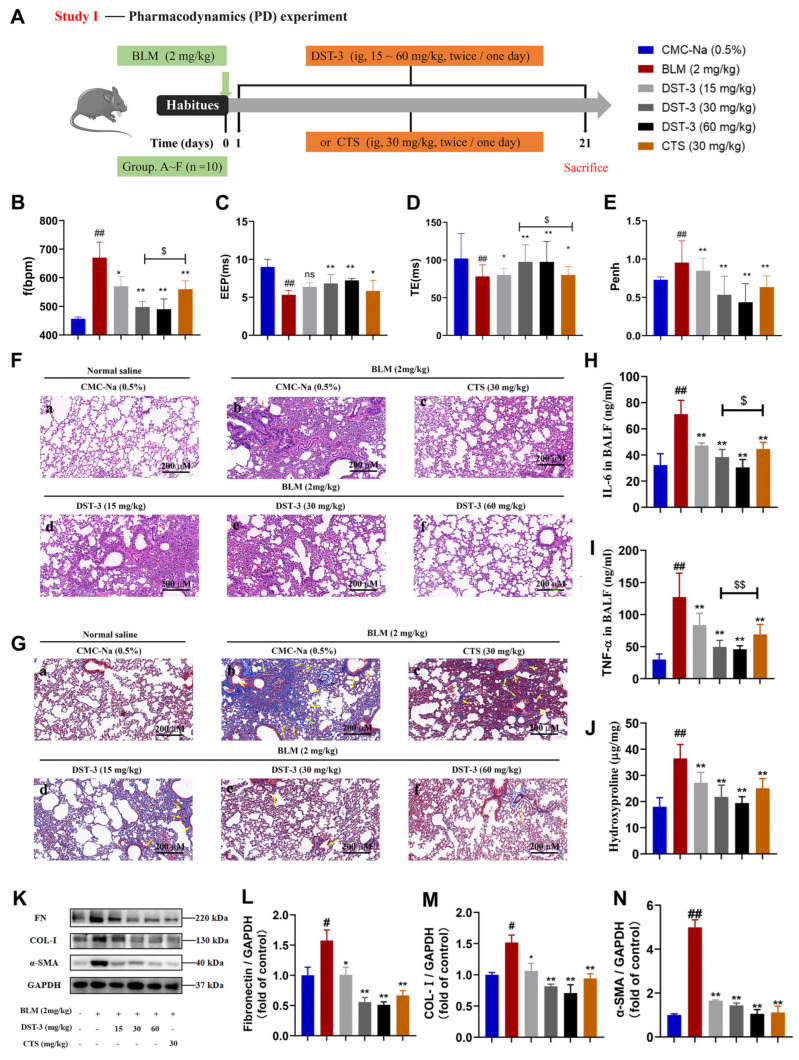
Effect of DST-3 treatment on BLM-induced pulmonary fibrosis in C57BL/6 mice. (**A**) Animal chart of the pharmacodynamic study (Study Ⅰ). (**B**–**E**) Measured lung function parameters, (**B**) frequency, (**C**) end-expiratory pause, (**D**) expiratory time, and (**E**) airway pressure narrow index. (**F**) H&E staining of lung tissue samples, scale = 200 μm. (**G**) Masson staining of lung tissue samples, scale = 200 μm. (**H**–**I**) Tumor necrosis factor-a (TNF-a) and Interleukin-6 (IL-6) secretion levels in bronchoalveolar lavage fluid (BALF) quantified by ELISA. (**J**) The HYP content in lung tissue. (**K**) The BLM-induced pulmonary inflammation and fibrosis reversed by DST-3 was detected by Western blot (fibronectin, type I collagen, and α-SMA). (**L**–**N**) Protein expression levels of (**L**) fibronectin, (**M**) type I collagen, (**N**) and α-SMA in lung tissue samples were analyzed by Western blotting. Abbreviations used in figures: Bleomycin, BLM; Cryptotanshinone, CTS; Carboxymethyl Cellulose Sodium, CMC-Na. In all figures: ^#^ *p* < 0.05, ^##^ *p* < 0.01 vs. control group; ** *p* < 0.05, * *p* < 0.01 vs. BLM group; ^$^ *p* < 0.05, ^$$^ *p* < 0.01 CTS (30 mg/kg) vs. DST-3 (30 mg/kg). ns: There was no statistical difference.

To evaluate the therapeutic effect of DST-3 on pulmonary fibrosis, we first established a BLM-induced pulmonary fibrosis model in C57BL/6 mice (single intratracheal instillation, 2 mg/kg). BLM administration led to significant lung function impairment, including increased respiratory rate (f, Figure 1C) and airway resistance (Penh, Figure 1F), along with decreased maximum expiratory flow rate (EEP, Figure 1D) and end-expiratory space (TE, Figure 1E). Treatment with DST-3 (15–60 mg/kg) dose-dependently rescued these functional deficits, and compared to the same dose of CTS (30 mg/kg), DST-3 significantly improved lung function.

We next assessed histopathological changes to determine whether DST-3 could alleviate BLM-induced structural lung damage. HE and Masson trichrome staining (Figure 1G–H) showed that BLM caused inflammatory cell infiltration, alveolar structural disorder, alveolar wall thickening, and collagen deposition. Both DST-3 (15–60 mg/kg) and CTS (30 mg/kg) reduced inflammatory infiltration and fibrosis severity, with DST-3 showing stronger effects. Consistently, DST-3 also suppressed proinflammatory-cytokines IL-6 (Figure 1I) and TNF-α (Figure 1J) in bronchoalveolar lavage fluid, and decreased hydroxyproline (HYP) content in lung tissue (Figure 1K), outperforming CTS at the same dose.

Finally, we examined the expression of α-SMA and ECM proteins to evaluate myofibroblast activation. BLM markedly increased their expression, whereas DST-3 treatment attenuated this upregulation (Figure 1L–N). Collectively, these results demonstrate that DST-3 dose-dependently mitigates BLM-induced pulmonary inflammation and fibrosis in C57BL/6 mice, with greater efficacy than an equivalent dose of CTS.

### 3.2. DST-3 Prevented TGF-β1-Induced Pulmonary Fibrosis in MRC5

**Figure 2:**
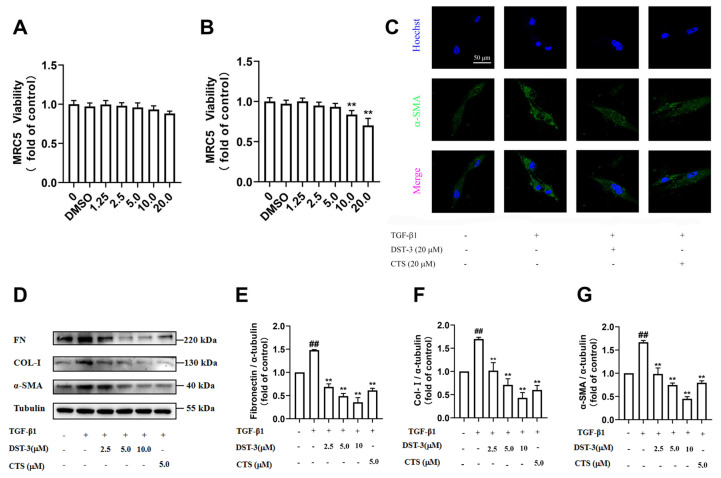
DST-3 prevents TGF-β1-induced pulmonary fibrosis in MRC5. (**A**) MRC5 cells were incubated with DST-3 (1.25, 2.5, 5, 10, 20 μM) for 48 h, and the cell viability ratio was determined by CCK8 method. (**B**) MRC5 cells were incubated with CTS (1.25, 2.5, 5, 10, 20 μM) for 48 h, and the cell viability ratio was determined by CCK8 method. (**C**) The expression of α-SMA was observed by immunofluorescence microscopy. Scale: 50 μm. Nuclei (blue, Hoechst 33342) and α-SMA (green) marking activated fibroblasts/myofibroblasts. Merged images reveal spatial colocalization of nuclear morphology and α-SMA expression. (**D**) MRC5 cells were treated with different concentrations of DST-3 for 48 h in the presence or absence of TGF-β1 (5 ng/mL) stimuli. The expression levels of (**E**) fibronectin, (**F**) type Ⅰ collagen, and (**G**) α-SMA protein were analyzed by Western blotting. Abbreviations used in figures: TGF-β1, Transforming Growth Factor Beta 1; DMSO, Dimethyl sulfoxide; Cryptotanshinone, CTS. In all figures: ^##^ *p* < 0.01 vs. control group; ** *p* < 0.01 vs. BLM group.

To investigate the protective effect of DST-3 against TGF-β1-induced pulmonary fibrosis in MRC5 cells, we first evaluated the cytotoxicity of DST-3 and CTS on MRC5 cells using the CCK8 assay. The results showed that DST-3 was still safe at 20 μM after 48 h treatment (Figure 2A), and the cell survival with CTS significantly decreased at 20 μM (Figure 2B). Based on these results, 5, 10, and 20 μM were selected as the optimal concentrations for in vitro experiments due to their favorable efficacy and low cytotoxicity. The results of immunofluorescent staining and Western blot results showed that both DST-3 and CTS could downregulate the overexpression of α-SMA and ECM proteins in MRC5 cells induced by TGF-β1 (Figure 2C,D). Quantitative analysis confirmed that DST-3 markedly attenuated TGF-β1-induced upregulation of Fn, Col-1, and α-SMA in MRC5 cells (Figure 2E–G). These results showed that DST-3 could significantly protect MRC5 cells from fibrosis induced by TGF-β1.

### 3.3. DST-3 Prevented Pulmonary Fibrosis by Directly Binding STAT3 to Inhibit JAK2/STAT3 Signaling Pathways

JAK2/STAT3 is an important advocate of inflammation and fibrosis pathways, and inhibition of JAK2/STAT3 can reduce alveolar inflammation and pulmonary fibrosis in IPF patients [39]. In this study, we investigated whether inhibition of STAT3 was involved in the protection of DST-3 against TGF-β1 or BLM-induced fibrosis.

**Figure 3:**
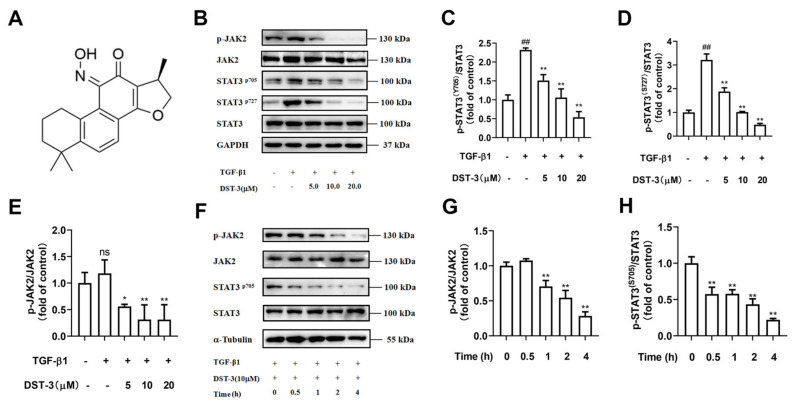
DST-3 inhibited TGF-β1-induced STAT3 activation in MRC5 cells and BLM-induced STAT3 activation in C57BL/6 mice. (**A**) DST-3 chemical formula. (**B**–**E**) MRC5 cells were treated with different concentrations of DST-3 (5–20 μM) for 2 h in the presence or absence of TGF-β1 stimulation. The protein expression levels of (**C**) p-STAT3^Tyr705^, (**D**) p-STAT3^Ser727^, and (**E**) p-JAK2^Tyr1007/1008^ were analyzed. (**F**–**H**) In the presence of TGF-β1 (5 ng/mL) stimulation, MRC5 cells were treated 0–4 h with the same concentration of DST-3 (10 μM). The protein expression levels of (**G**) p-STAT3^Tyr705^ and (**H**) p-JAK2^Tyr1007/1008^ at different time points were analyzed by Western blot. Abbreviations used in figures: TGF-β1, Transforming Growth Factor Beta 1; Bleomycin, BLM; Cryptotanshinone, CTS. In all figures, ns indicates no significant difference, ^##^ *p* < 0.01 vs. control group; ** *p* < 0.05, * *p* < 0.01 vs. TGF-β1 or BLM group.

Our results show that STAT3 (Tyr705 and Ser727) of MRC5 cells were significantly phosphorylated after 2 h treatment with TGF-β1 (Figure 3A–E). To determine whether DST-3 modulates STAT3 phosphorylation through inhibition of JAK2 kinase activity, we performed a time-course experiment in TGF-β1-treated MRC5 cells, administering DST-3 at 0, 0.5, 1, 2, and 4 h. Notably, p-JAK2 levels remained largely unchanged throughout the time course, whereas p-STAT3 was significantly inhibited at early time points (Figure 3F–H). These results suggest that DST-3 may directly target STAT3 to suppress its phosphorylation, independent of JAK2 inhibition

**Figure 4:**
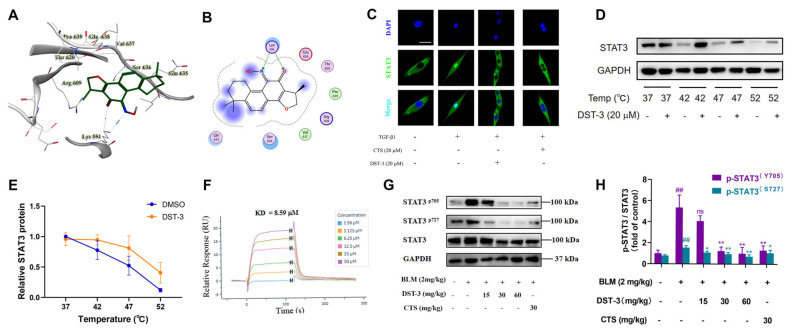
DST-3 inhibited TGF-β1 induced STAT3 activation in MRC5 cells and BLM-induced STAT3 activation in C57BL/6 mice. (**A**,**B**) Computational molecular docking analysis of DST-3 binding interactions with STAT3 (PDB: 6NUQ). The dark green molecule represents that Figure A is a 3D graph and Figure B is a 2D planar graph of DST-3. (**C**) The subcellular localization of STAT3 was observed by immunofluorescence microscopy. Scale bar: 50 mm. (**D**,**E**) Melting curves of STAT3 protein in the cellular thermal shift assay in MRC5 cells treated with DST-3 or DMSO for 1 h. Panel M shows quantification of STAT3 protein and temperature points. (**F**) SPR analysis of DST-3 binding to STAT3. (**G**,**H**) The protein expression levels of p-STAT3^Tyr705^ and p-STAT3^Ser727^ in lung tissue samples were analyzed. Abbreviations used in figures: TGF-β1, Transforming Growth Factor Beta 1; Bleomycin, BLM; Cryptotanshinone, CTS. In all figures, ns indicates no significant difference, ^##^ *p* < 0.01 vs. control group; ** *p* < 0.05, * *p* < 0.01 vs. TGF-β1 or BLM group.

To elucidate the molecular mechanism by which DST-3 directly inhibits STAT3, we performed molecular docking simulations. Molecular docking simulations revealed that DST-3 binds to the phosphotyrosine (PY) pocket within the SH2 domain of STAT3 (Figure 4A,B). This pocket functions as a phosphotyrosine-binding site that mediates STAT3 dimerization and conformational changes, playing a critical role in phosphorylation-dependent activation. Thus, the formation of a hydrogen bond between DST-3 and Lys591 may impair the binding capacity of the SH2 domain, thereby destabilizing STAT3 conformation and altering its phosphorylation status. We also found that DST-3 could stabilize STAT3 protein at 37–52 °C through the Cellular thermal shift assay (Figure 4C–E), which proved the binding of DST-3 to STAT3 protein. Meanwhile, surface plasmon resonance (SPR) experiments showed that DST-3 responded to the STAT3-loaded chip with an affinity of 8.59 μM (Figure 4F). In addition, we demonstrated that DST-3 inhibited the levels of p-STAT3 (Tyr705 and Ser727) in C57BL/6 mice with BLM-induced pulmonary fibrosis (Figure 4G,H).

### 3.4. DST-3 Prevented TGF/Smad-Mediated Pulmonary Fibrosis

**Figure 5:**
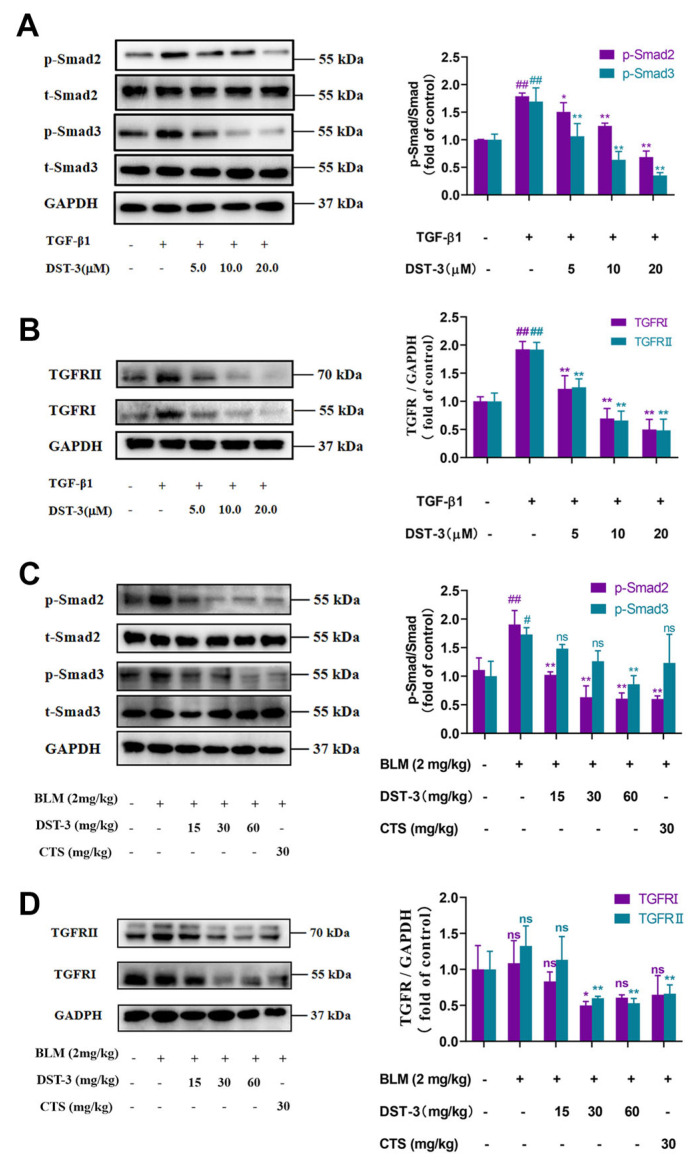
DST-3 prevented pulmonary fibrosis by suppressing TGF/Smad signaling pathways. (**A**) MRC5 cells were treated with different concentrations of DST-3 for 0.5 h in the presence or absence of TGF-β1 (5 ng/mL) stimuli. The protein expression levels of p-Smad2/3 were analyzed by Western blotting. (**B**) MRC5 cells were treated with different concentrations of DST-3 for 48 h in the presence or absence of TGF-β1 (5 ng/mL) stimuli. The protein expression levels of TGF RI and TGF RⅡ were analyzed by Western blotting. (**C**) Lung tissue from C57BL/6 mice. The protein expression levels of p-Smad2/3 were analyzed by Western blotting. (**D**) Lung tissue from C57BL/6 mice. The protein expression levels of TGF RI and TGF RⅡ were analyzed by Western blotting. Abbreviations used in figures: TGF-β1, Transforming Growth Factor Beta 1; Bleomycin, BLM; Cryptotanshinone, CTS. In all figures: ^#^ *p* < 0.05, ^##^ *p* < 0.01 vs. control group; ** *p* < 0.05, * *p* < 0.01 vs. TGF-β1 or BLM group. ns: There was no statistical difference.

To determine whether DST-3 modulates pulmonary fibrosis via the TGF/Smad pathway, we first examined the expression of TGF-β receptors and Smad2/3 phosphorylation in MRC5 cells. The imbalance of homeostasis in the TGF/Smad pathway is an important cause of fibrosis [40]. TGF-β1 promotes the fibrosis process by directly activating the Smad2/3 to trigger the overexpression of the fibrosis genes, including fibroblast to myofibroblast transformation, namely the overexpression of α-SMA and the overdeposition of ECM [41]. To investigate whether DST-3 influences pulmonary fibrosis through TGF/Smad, in the presence or absence of TGF−β1, we found that the protein levels of transforming growth factor-β receptor type I (TGF-β RI) and transforming growth factor-beta receptor type II (TGF-β RII) were significantly upregulated (Figure 5A), and DST-3 reversed this upregulation in the presence of TGF-β1 for 48 h (Figure 5B). At the same time, the levels of p-Smad2/3 were significantly upregulated after 0.5 h treatment with TGF-β1. DST-3 also inhibited the levels of p-Smad2/3 in a dose-dependent manner (Figure 5A). Similarly, DST-3 significantly reduced the elevated p-Smad2/3 levels in BLM-induced pulmonary fibrosis in C57BL/6 mice (Figure 5C). It also downregulated both types of TGFR as in cells (Figure 5D).

### 3.5. Validation of the HPLC-MS/MS Method for Simultaneous Quantitative Analysis of DST-3

To establish a suitable quantitative analysis method for DST-3, we tried different proportions of mobile phase and compared gradient elution and equal degree elution. DST-3 and LTD exhibited excellent separation and peak shape, with complete elution achieved in 6 min using a methanol: 1.0% formic acid water solution (90:10, *v*/*v*). In addition, mass spectrometry parameters were optimized to enhance ionization efficiency, ensuring DST-3 and LTD were fully decomposed into stable product ions. For better quantitative analysis, we selected more suitable DST-3 collision conditions and product ions to obtain higher response values and good peak shape.

To evaluate our established HPLC-MS/MS quantitative assay, specificity, linearity, lower limit of quantitation (LLOQ), precision, accuracy, sample stability, matrix effects, and recovery were validated (Supplementary Material S3). Based on blank plasma, tissue samples, low-concentration DST-3 QC samples, and plasma samples following oral administration of DST-3 (60 mg/kg), chromatography results indicated that the peak times for DST-3 and the internal standard LTD were approximately 4.15 min and 1.76 min, respectively. The internal standard and endogenous matter did not interfere with DST-3 detection, indicating that the proposed method is highly specific. DST-3 plasma and tissue samples also showed good linearity and correlation coefficients (R^2^ > 0.99) with a linear range of 2–500 ng/mL, with RE of 10.0% and RSD of 9.0% for six LLOQ samples. To verify the accuracy of the method, we analyzed plasma and tissue QC samples from the same batch on three consecutive days. The RE and RSD of low QC samples were −2.8%~5.6% or −1.2%~8.9%, and −1.0%~8.6% or 4.9%~11.4%, respectively. The RE and RSD of medium QC samples were −1.0%~−6.7% or −4.7%~−0.8%, and 4.6%~14.4% or 7.9%~14.5%, respectively. The RE and RSD of high QC samples were −5.4%~4.5% or −0.7%~2.8% and 6.1%~14.1% or 6.9%~11.1%, respectively. All RE were in the range of −20% to 20% and all RSD were less than 20% and in line with the guidelines. The samples were stored at room temperature for 48 h or frozen at −80 °C for one month before being analyzed. The RE and RSD of low QC samples were −19.2%~3.5% or −12.4%~8.5%, and 7.3%~13.8% or 5.0%~16.9%, respectively. The RE and RSD of high QC samples were −9.8%~10.5% or −8.2%~8.2%, and 2.7%~11.0% or 4.5%~18.5%, respectively. All RE were in the range of −20% to 20% and all RSD were less than 20%. In addition, we analyzed low QC samples prepared in plasma and tissue matrices from the same batch. The average ratio of peak responses (tissue/plasma) was approximately 0.96–1.02, with RSD values ranging from −3.4% to 4.1% and RE values from −3.6% to 0.1%, indicating a negligible matrix effect. More detailed data are provided in Supplementary Material S3.

In summary, we establish a sufficiently sensitive, stable, and efficient HPLC-MS/MS method. The method provides sufficient exclusivity and biological sensitivity to be used for routine analysis and pharmacokinetic study in vivo.

### 3.6. Pharmacokinetics of DST-3

**Figure 6:**
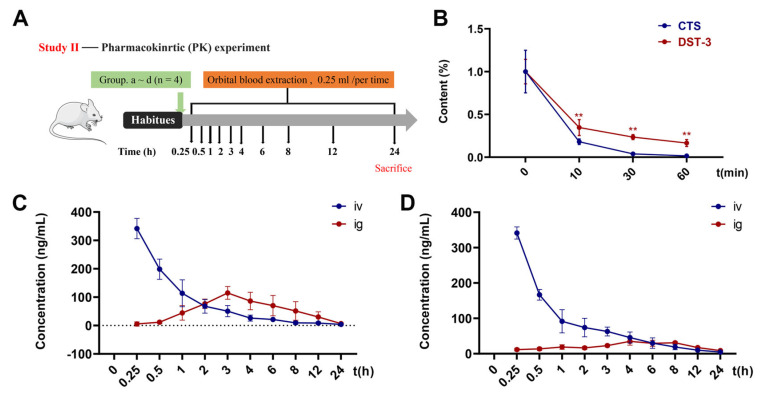
Pharmacokinetic study of DST-3 in SD rats. (**A**) Animal chart of the pharmacokinetic study (Study Ⅱ). (**B**) Stability of DST-3 and CTS liver microsomes (** *p* < 0.05). (**C**) Mean plasma concentration-time curves of SD rats at single DST-3 administration in SD rats. (**D**) Mean plasma concentration-time curves of SD rats at single CTS administration in SD rats.

The verified method was successfully applied to SD rats after a single intravenous injection of 6 mg/kg or oral administration of 60 mg/kg DST-3. The DST-3 and CTS concentration in plasma was measured at different time points (Supplementary Material S3). According to the data, the mean plasma concentration–time curve was obtained (Figure 6C,D). The main pharmacokinetic parameters of intravenous injection or gavage based on the non-atrioventricular model were calculated as follows (Table 3). The absolute bioavailability of DST-3 was 16.48%, which was significantly higher than that of CTS (8.56%, absolute bioavailability = AUC_ig_/AUC_iv_ × 100%).

The system was activated after the administration of NRS. It can be seen from the rapid metabolism of DST-3 and CTS at 10 min. At 10 min, 30 min, and 60 min after activation of the hepatic microsomal system, the stability of DST-3 in the presence of hepatic microsomes was significantly higher than that of CTS. In addition, the metabolic rate of DST-3 exceeded 80% after 60 min, meaning the absence of liver microsomal accumulation, as shown in Figure 6B.

### 3.7. Tissue Distribution of DST-3

**Figure 7:**
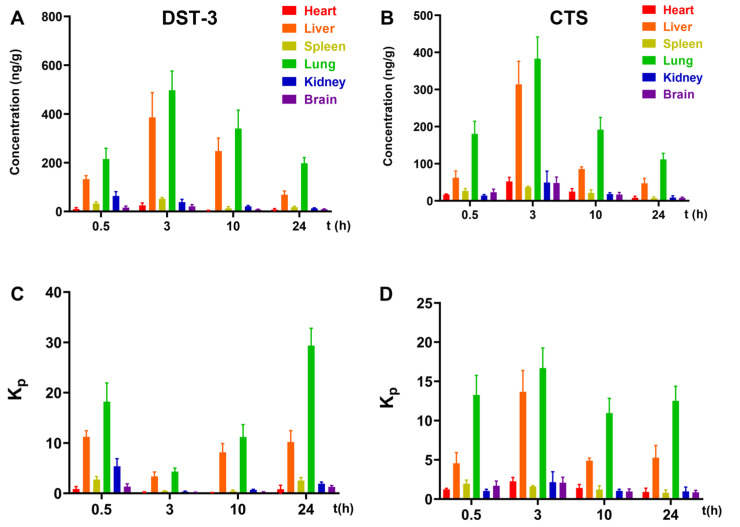
Tissue distribution study of DST-3 in SD rats. (**A**) The concentration of DST-3 in tissues. (**B**) The concentration of CTS in tissues. (**C**) Tissue-to-plasma partition coefficient of DST-3 in tissues. (**D**) Tissue-to-plasma partition coefficient of CTS in tissues. Data are presented as mean ± SD (*n* = 4).

The concentrations of DST-3 or CTS in the heart, liver, spleen, lung, kidney, and brain were measured at different times after a single dose of 60 mg/kg oral administration. DST-3 is widely distributed in all tissues. Revealing the following trend of 0.5 h lung > liver > kidney > spleen > brain > heart; 3 h lung > liver > spleen > kidney > heart > brain; 10 h lung > liver > kidney > spleen > brain > heart; 24 h lung > liver > spleen > kidney > heart > brain. As shown in Figure 7A,B, DST-3 showed higher absolute lung concentrations than CTS. However, this may be partly attributable to its higher plasma exposure. To minimize the confounding effect of plasma exposure, we further compared the tissue-to-plasma partition coefficients (Kp, Figure 7C,D), which confirmed that the lung Kp of DST-3 was not only higher than that of CTS but also higher than Kp values in most other tissues (particularly at 24 h), indicating a preferential distribution of DST-3 into the lung independent of plasma exposure differences.

## 4. Discussion

Idiopathic pulmonary fibrosis continues to be a challenging disease globally, lacking a known cause and a complete cure despite extensive research efforts over the years. In this study, we found that DST-3 markedly improved the pharmacokinetic profile and lung exposure compared to its parent compound CTS, supporting its potential as a more effective therapeutic for pulmonary fibrosis. Although pirfenidone and nintedanib are currently the only FDA-approved drugs for IPF and can slow disease progression [42,43], their clinical application is often limited by adverse effects, including gastrointestinal reactions, photosensitivity, and hepatotoxicity [5,44]. Our previous work demonstrated that CTS effectively attenuates bleomycin-induced pulmonary fibrosis in SD rats at doses much lower than pirfenidone, with faster absorption and higher systemic and lung exposure [30,45]. However, CTS is a substrate of P-glycoprotein (PgP), resulting in very low oral bioavailability (2.05%) [31]. By contrast, DST-3 reached peak concentration more rapidly and achieved higher maximum concentration and area under the curve at the same dose, as shown by our newly established HPLC-MS/MS method, indicating significantly improved absorption and bioavailability. The calculated absolute bioavailability is 16.48%. Higher bioavailability typically enables lower dosing, potentially widening the therapeutic window and increasing clinical applicability. However, the net effect depends on balancing enhanced therapeutic benefits against possible side effects. Tissue distribution studies had also shown that exposure to DST-3 in the lungs was significantly higher than that to the same dose of CTS. DST-3 may have greater potential as a treatment for pulmonary fibrosis compared to CTS.

BLM is an alkaline glycopeptide antibiotic produced by Streptomyces rotans [46], which is used in the early treatment of cancer [47]. However, the strong pulmonary toxicity of BLM can lead to an extremely severe inflammatory response and pulmonary fibrosis in experimental animals receiving an endotracheal drip of BLM [48]. Therefore, BLM is still one of the most reasonable and commonly used modeling methods for studying pulmonary fibrosis [49]. Our results showed that DST-3 alleviated BLM-induced lung function decline, pathological changes, inflammatory factor release, and collagen deposition in C57BL/6 mice in a dose-dependent manner at 15–60 mg/kg with no toxic side effects at high doses (60 mg/kg). Compared to the same dose of CTS, DST-3 has a more obvious improvement effect on pulmonary fibrosis.

TGF-β1 plays an important role in the progression of physiological healing and fibrosis [50]. TGF-β1 conducts fibrotic processes through the non-Smad pathway [51], such as involving enhancement of p-STAT3 and transseating into the nucleus after activation of JAK2 [52]. In recent years, different compounds have been reported to improve pulmonary fibrosis by inhibiting the phosphorylation of STAT3 [39,53]. In this study, we found that DST-3 inhibited the p-JAK2 and abnormally upregulated p-STAT3^Tyr705/Ser727^ in the presence of TGF-β1. Furthermore, DST-3 can also bypass JAK2 and directly bind to STAT3, inhibiting its phosphorylation and reducing its presence in the nucleus. STAT3 has three pockets in the SH2 domain that can be targeted by small molecules. Embedding small molecules into protein pockets affects p-STAT3^Tyr705/Ser727^ and further affects STAT3 dimerization [54]. Our experiments showed that the oxime and keto groups of DST-3 targeted Lys591 in the PY pocket of the SH2 domain of STAT3 and formed two hydrogen bonds, which may be the cause of the direct influence of DST-3 on STAT3. In addition, we demonstrated that DST-3 reduced p-STAT3^Tyr705/Ser727^ in mice with BLM-induced pulmonary fibrosis. Inhibiting the JAK2/STAT3 signaling pathway is believed to suppress the TGF/Smad pathway [55]. TGF-β1 mediates the occurrence and development of lung, kidney, and liver fibrosis through the classical TGF/Smad pathway [56]. This is because TGF-β1 further promotes fibrosis by inducing phosphorylation of Ser255/Ser213 in the Smad2/3 junction region [57]. Our results suggested that DST-3 can effectively inhibit p-Smad2^Ser255^ and p-Smad3^Ser213^ in vivo and in vitro. The number of TGF type Ⅰ and Ⅱ receptors also increases in the progression of pulmonary fibrosis and is involved in the production of ECM [58]. Our results showed that DST-3 reduced the increase in the number of both types of TGF receptors in MRC5 cells induced by TGF-β1, although this effect was not significant in C57BL/6 mice with BLM-induced pulmonary fibrosis. Thus, DST-3 inhibits JAK2/STAT3 signaling by directly binding STAT3 and inhibiting the TGF/Smad pathway to reduce fibrosis.

Natural drugs play an important role with multiple targets, and pulmonary fibrosis is a multifactorial disease. DST-3, a structural modification of CTS, may target several pathways in vivo, leading to strong anti-fibrotic effects. In summary, we found that a novel modified CTS, DST-3, was capable of treating BLM-induced fibrosis in C57BL/6 mice or TGF-β1-induced fibrosis in MRC5. This therapeutic effect was achieved by directly targeting STAT3 to inhibit the STAT3 pathway and suppress the TGF/Smad pathway. In addition, we also established a sensitive, efficient, and stable HPLC-MS/MS quantitative analysis method for DST-3, and verified the feasibility of this method. The results showed that oral DST-3 significantly improved lung exposure and bioavailability compared to oral CTS at the same dose. This indicates that DST-3 is a more promising candidate compound for the treatment of pulmonary fibrosis and provides an important basis for further understanding of the structure-activity relationship of CTS and its influencing factors on pharmacokinetics. Taken together, our results demonstrate that DST-3 exhibits favorable pharmacokinetic properties, effective tissue distribution (especially in the lungs), and significant anti-fibrotic activity through inhibition of STAT3 and Smad signaling pathways. These findings suggest that DST-3 may serve as a promising lead compound for the development of novel therapeutics targeting pulmonary fibrosis. With its improved oral bioavailability and pharmacological efficacy, DST-3 has translational potential to advance toward preclinical development and potentially clinical evaluation, pending further safety and toxicological studies.

## Figures and Tables

**Table 1:** Ion pairs and collision energy of DST-3 and LTD.

Compounds	Precursor Ion (*m/z*)	Product Ion (*m/z*)	Mode	CE (V)
DST-3	312	294	Positive	18
CTS	297	251	Positive	21
Loratadine	383	266	Positive	31

**Table 2:** Summary of mass spectrometry parameters used for DST-3 quantification.

Parameter	Setting
Ionization mode	Electrospray Ionization (ESI)
Scan mode	Selected Reaction Monitoring (SRM)
Polarity	Positive ion mode
Spray voltage	3000 V
Sheath gas pressure	50 psi
Auxiliary gas pressure	10 psi
Capillary temperature	350 °C
Peak width (Q1/Q3)	20.0 s
Collision gas (argon) pressure	1.9 mtorr
Scan width	0.7 *m/z*
Dwell time (scan time)	0.1 s

**Table 3:** Main pharmacokinetic parameters of single administration in SD rats (*n* = 4, ** *p* < 0.05 vs. control group).

PK Parameters	DST-3 (Mean ± SD)		CTS (Mean ± SD)	
	iv	ig	iv	ig
T_max_ (h)	/	3.000 ± 0.0000	/	4.500 ± 1.000
C_max_ (ng/mL)	341.6 ± 31.00	114.9 ± 22.50 **	341.3 ±14.90	39.50 ± 6.800
AUC_0–t_ (ng/mL·h)	560.5 ± 11.50	893.0 ± 427.7 **	630.6 ± 157.0	457.5 ± 22.80
AUC_0–∞_ (ng/mL·h)	569.1 ± 7.600	938.0 ± 411.6 **	671.0 ± 167.8	574.1 ± 51.00
CL/F (L/h)	10.50 ± 0.1000	71.90 ± 24.90 **	8.94 ± 2.100	104.5 ± 10.40
V/F (L)	56.10 ± 31.70	466.5 ± 236.4 **	87.70 ± 59.30	1391 ± 382.0
t_1/2_ (h)	3.700 ± 2.100	4.500 ± 2.000 **	6.800 ± 3.700	9.300 ± 3.000
MRT_0–t_ (h)	4.200 ± 0.7000	6.900 ± 1.200 **	5.100 ± 0.3000	15.70 ± 2.800

Abbreviations: AUC, area under the concentration–time curve (ng·h/mL); t_1_/_2_, elimination half-life (h); CL, clearance (L/h/kg); Vd, apparent volume of distribution (L/kg); MRT, mean residence time (h).

## Data Availability

Data is contained in the paper.

## Supplementary Materials

The following supporting information can be downloaded at: https://www.mdpi.com/article/10.3390/pharmaceutics17101307/s1, Supplement Material S1—compound DST-3; Supplement Material S2—Antibody; Supplement Material S3—Validation of methodology.

## Abbreviations

The following abbreviations are used in this manuscript:

CTS	Cryptotanshinone
BLM	Bleomycin
IPF	Idiopathic pulmonary fibrosis
ECM	Extracellular matrix
STAT	Signal transducers and activators of transcription
HE	hematoxylin-eosin
HYP	hydroxyproline
SPR	Surface plasmon resonance
WB	Western blot
ELISA	Enzyme-linked immunosorbent assay
TBST	Tris-Buffered Saline with Tween 20
QC	Quality control
LLOQ	Lower limit of quantitation
Kp	Tissue-to-plasma partition coefficients
CMC-Na	Sodium carboxymethyl cellulose
SD	Sprague-Dawley
