# Supplementary material for: DST-3, a Novel Modified Cryptotanshinone, Protects Against Pulmonary Fibrosis via Inhibiting STAT3/Smad Signaling Pathway and Improves Bioavailability
Source: Pharmaceutics. 2025 Oct 8;17(10):1307. doi: 10.3390/pharmaceutics17101307 (PMC12566936; doi:10.3390/pharmaceutics17101307)
Supplement: Supplementary file 1 [file pharmaceutics-17-01307-s001.zip › Supplement Material S1 - compound DST-3.pdf]

Chemical synthesis of DST-3

In a 500 mL flask, 2.0 g cryptotanshinone was added, suspended with 200 mL anhydrous ethanol, followed by 5.0 g hydroxylamine hydrochloride and 5.0 g anhydrous sodium carbonate. The reaction was carried out overnight at room temperature. After the reaction was completed, the solvent was removed by vacuum distillation. The reaction mixture was suspended in 100 mL water and extracted for 3 times with 100 mL ethyl acetate. The organic layer was incorporated, and an appropriate amount of anhydrous sodium sulfate was added for drying. The crude product was purified by silica gel column chromatography [V (petroleum ether) : V (ethyl acetate) = 1:0 → 200:1] to obtain brown solid product, which was recrystallized by dichloromethane/methanol system to obtain red massive solid compound, 980 mg, with a yield of 47%.

NMR data of DST-3

^{1}H NMR (400 MHz, $CDCl_3$), δ: 7.62 (d, *J*=8.3 Hz, 1H), 7.51 (d, *J*=8.3 Hz, 1H), 4.93 (t, *J*=9.4 Hz, 1H), 4.41 (dd, *J*=5.8, 9.4 Hz, 1H), 3.66 (m, 1H), 3.16 (t, *J*=6.1 Hz, 2H), 1.74 (m, 2H), 1.69 (m, 2H), 1.39 (d, *J*=6.8 Hz, 3H), 1.34 (s, 3H), 1.33 (s, 3H). ^{13}C NMR (100 MHz, $CDCl_3$), δ: 178.8, 172.2, 152.3, 148.7, 137.8, 130.5, 127.8, 121.9, 120.1, 116.3, 81.9, 38.4, 35.4, 34.1, 33.1, 32.3, 32.3, 20.1, 18.7. HRESI-MS *m*/*z*: $[M+Na]^+$. The theoretical value is 334.1438, and the measured value is 334.1434.

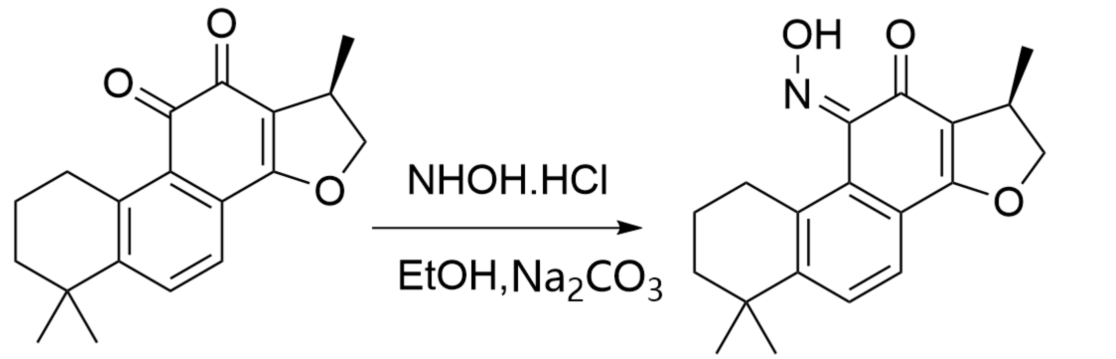

Figure S1-1 Diagram of chemical synthesis of compound DST-3.

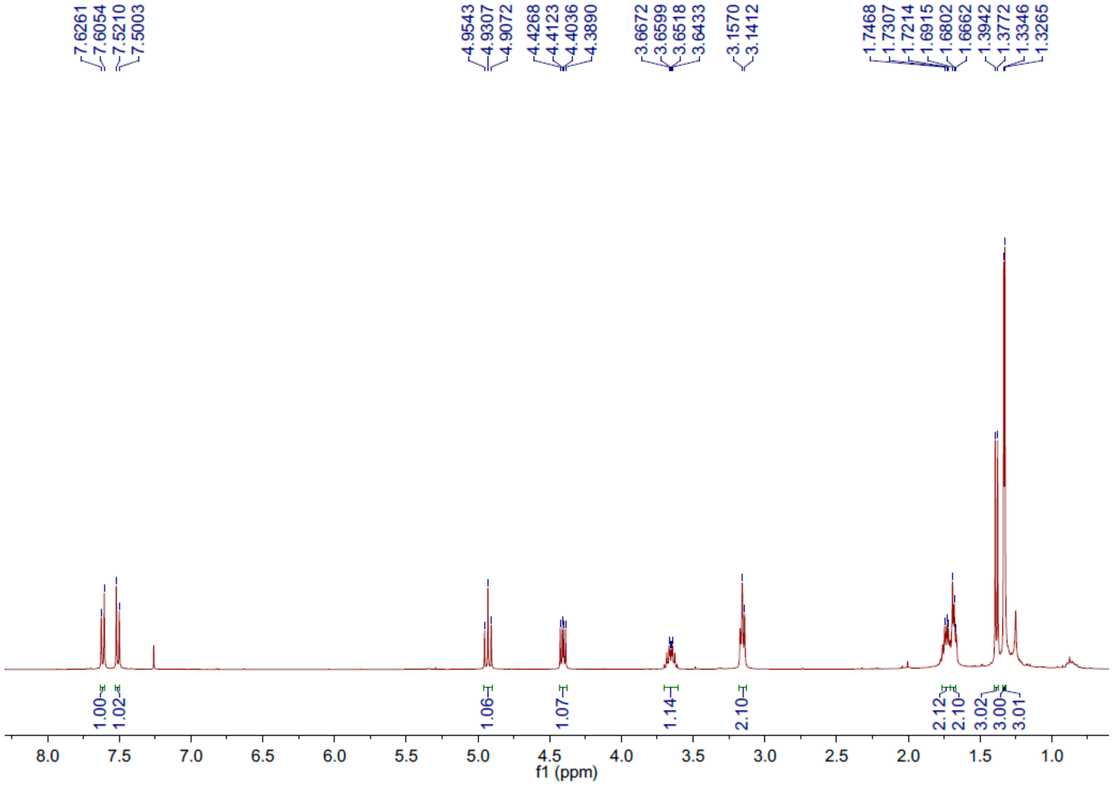

Figure S1-2 ^{1}H NMR (400 MHz, $CDCl_3$) spectrum of compound DST-3.

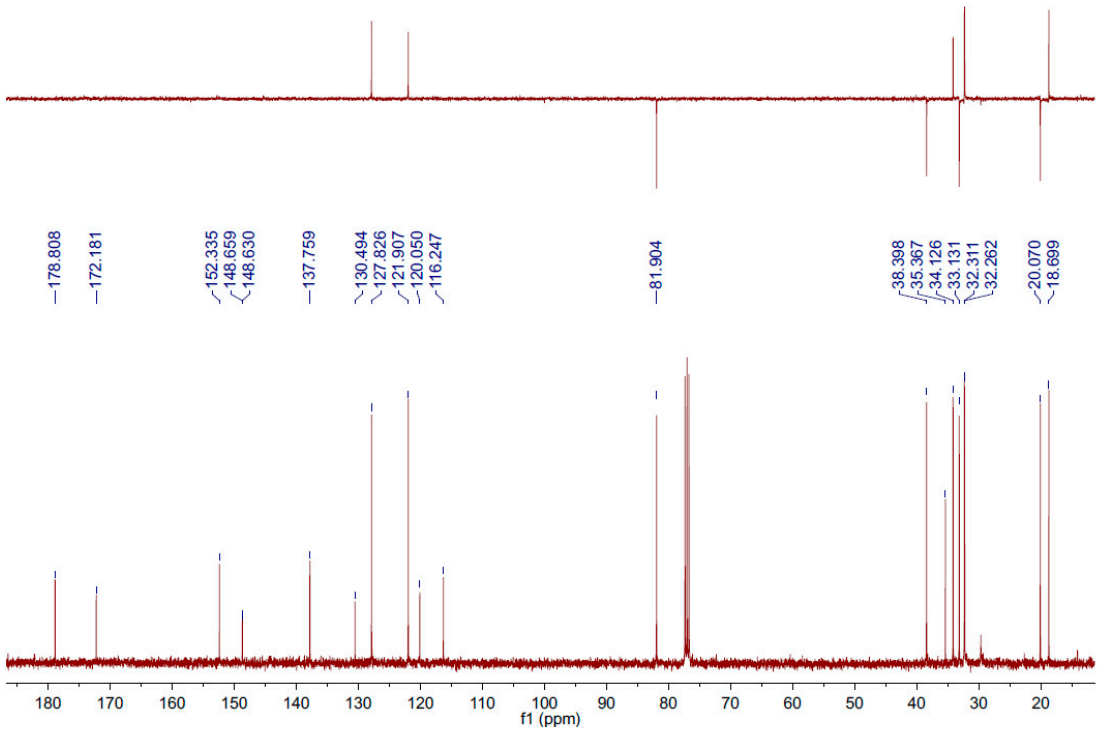

Figure S1-3 ^{13}C NMR and DEPT (125 MHz, $CDCl_3$) spectra of compound DST-3.

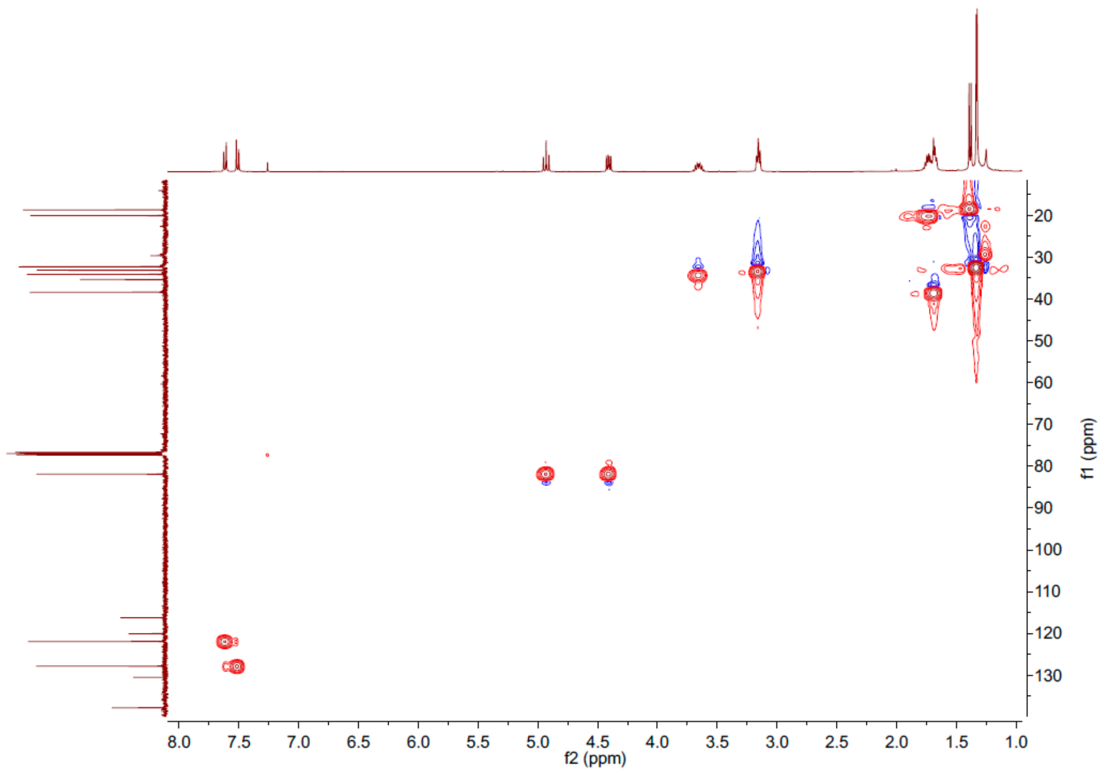

Figure S1-4 HSQC (400 MHz, $CDCl_3$) spectrum of compound DST-3.

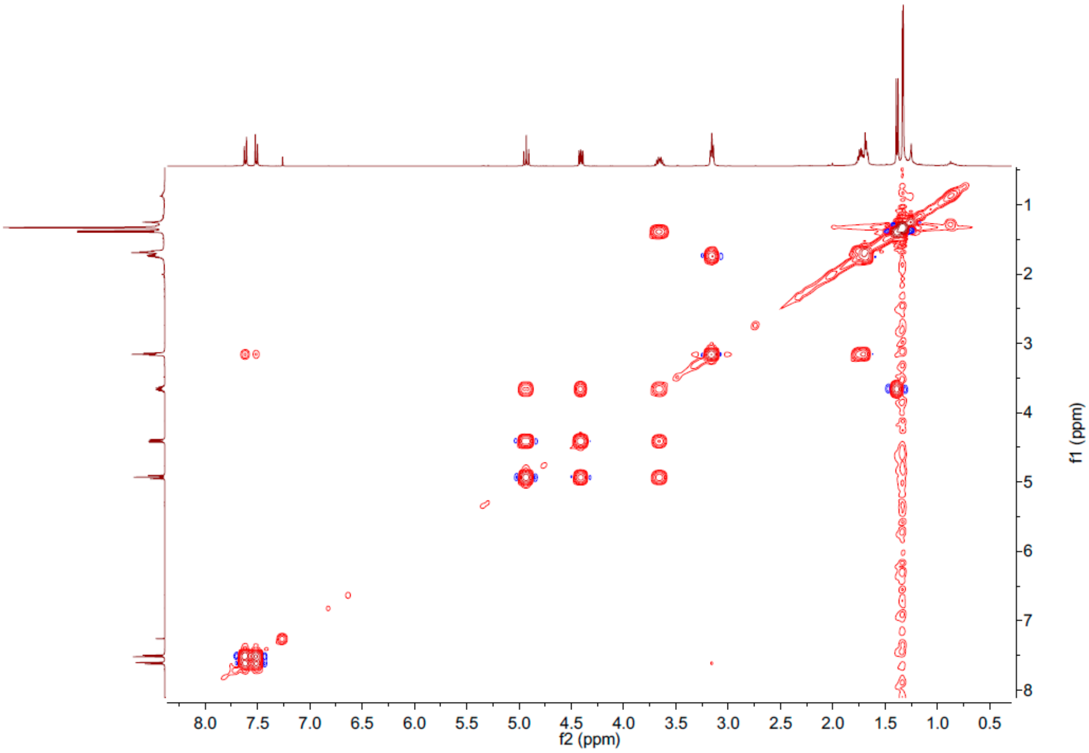

Figure S1-5 ^{1}H–^{1}H COSY (400 MHz, $CDCl_3$) spectrum of compound DST-3.

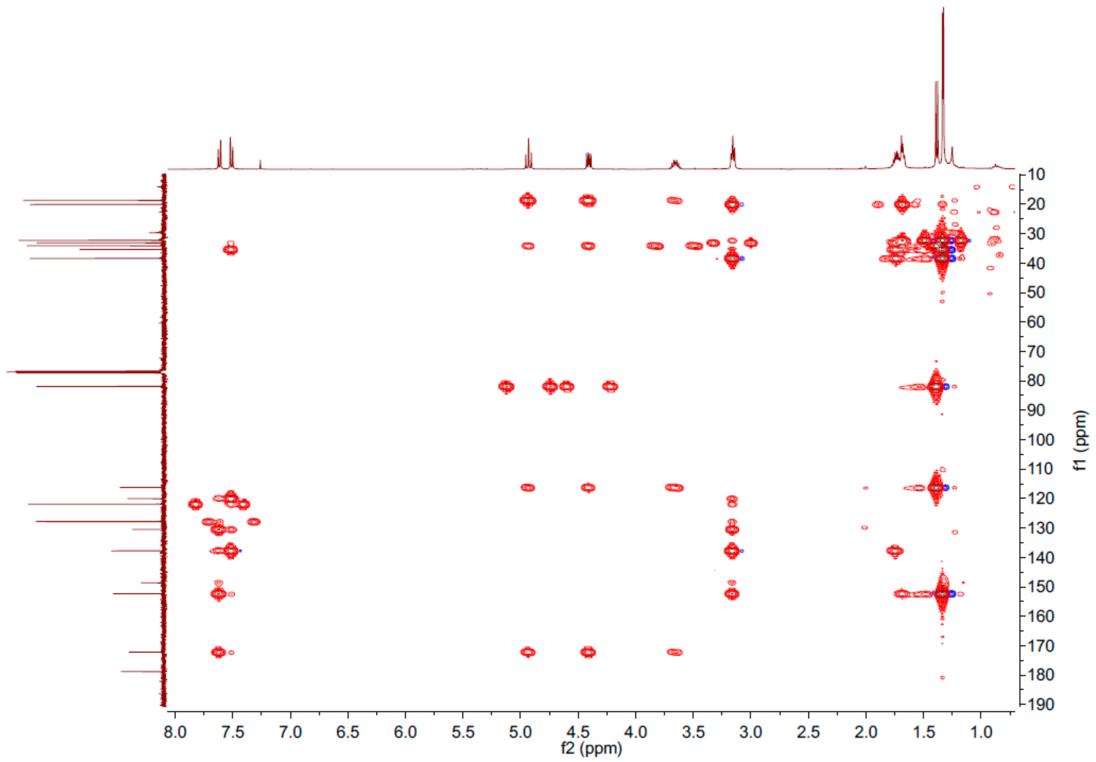

Figure S1-6 HMBC (400 MHz, CDCl3) spectrum of compound DST-3.

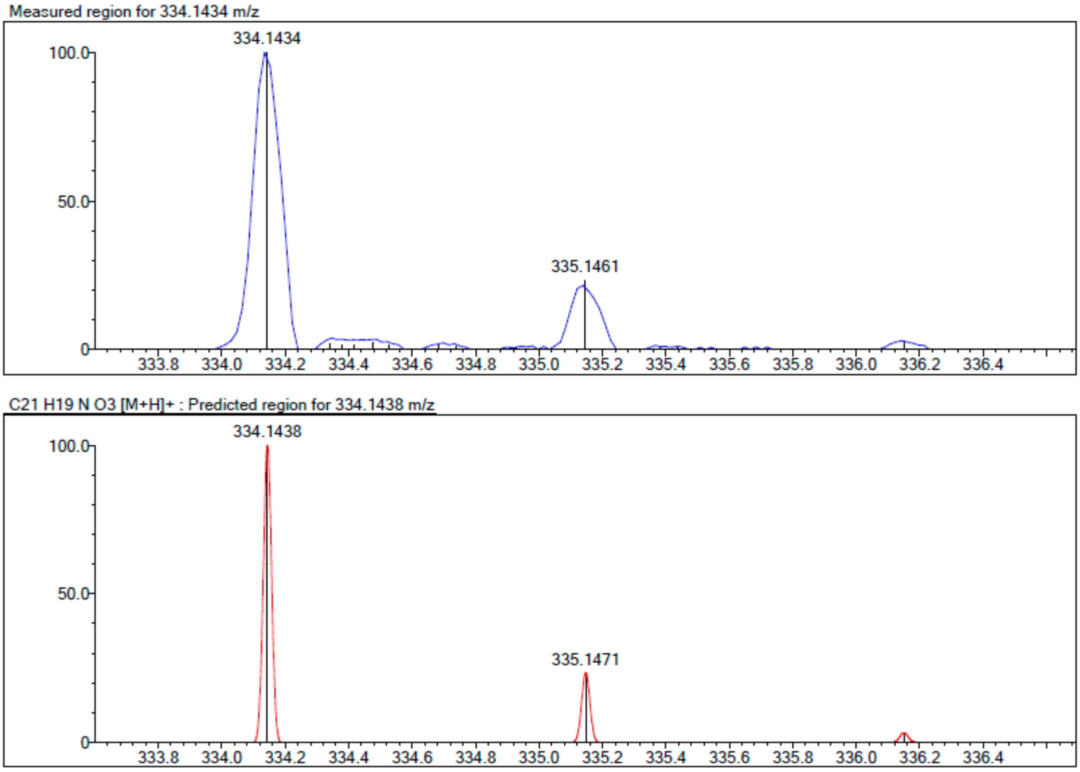

Rank	Score	Formula (M)	Ion	Meas. m/z	Pred. m/z	Df. (mDa)	Df. (ppm)	Iso	DBE
1	70.49	C21 H19 N O3	[M+H]+	334.1434	334.1438	-0.4	-1.20	70.84	13.0

Figure S1-7 HRESI-MS data of compound DST-3.

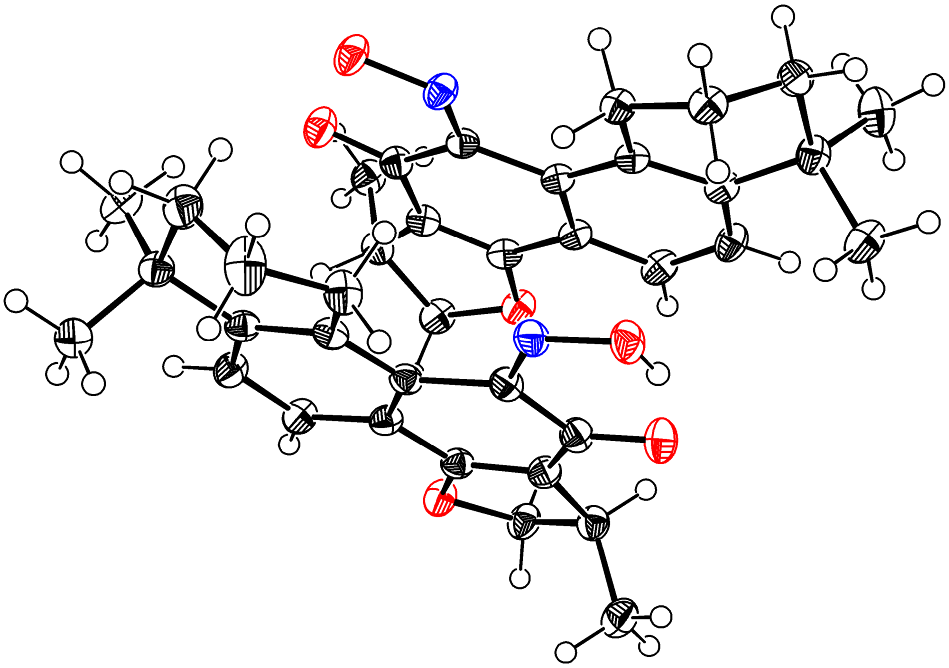

Figure S1-8 Single crystal diffraction image of DST-3.

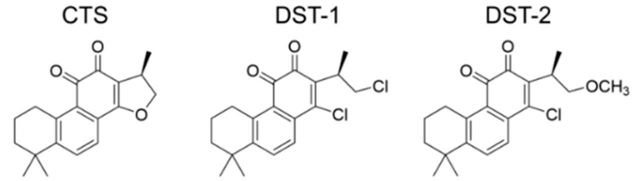

Figure S1-9 Chemical structures of CTS and its derivatives.

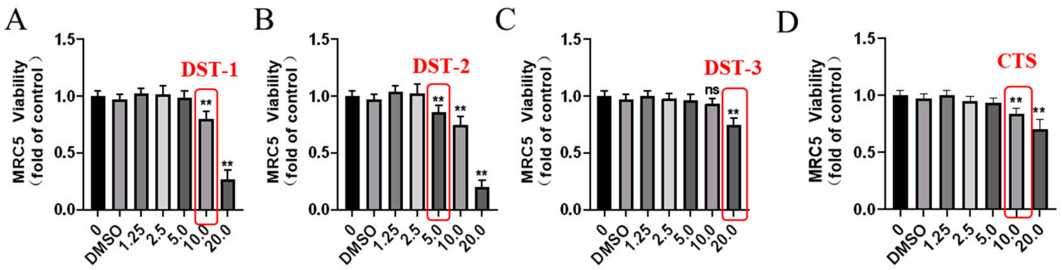

Figure S1-10 Preliminary cytotoxicity screening of different CTS derivatives. (A) DST-1, (B) DST-2, (C) DST-3, (D) CTS.
