# Supplementary material for: DST-3, a Novel Modified Cryptotanshinone, Protects Against Pulmonary Fibrosis via Inhibiting STAT3/Smad Signaling Pathway and Improves Bioavailability
Source: Pharmaceutics. 2025 Oct 8;17(10):1307. doi: 10.3390/pharmaceutics17101307 (PMC12566936; doi:10.3390/pharmaceutics17101307)
Supplement: Supplementary file 1 [file pharmaceutics-17-01307-s001.zip › Supplement Material S2 - Antibody.pdf]

Table S2-1. Information on antibodies

	Antibody	Brand	Art.No.
Primary antibodies	Phospho-Stat3(Ser727)	Cell Signaling Technology	9134T
	STAT3	Proteintech Group	10253-2-AP
	Phospho-Stat3 (Tyr705)	Cell Signaling Technology	9145T
	Recombinanty Anti-Smad2 (phospho S255)	Abcam	ab188334
	Recombinanty Anti-Smad2	Abcam	ab40855
	Phospho-Smad3 (Ser213)	Affinity Biosciences	AF3366
	Recombinanty Anti-Smad3	Abcam	ab40854
	GAPDH	huabio	ET1601-4-T
	α-Tubulin	Abcam	ab7291
	TGF beta Receptor I	Abcam	ab235578
	TGF beta Receptor II	Abcam	ab184948
	smooth muscle actin	Proteintech	14395-1-AP
	Fibronectin Polyclonal	Proteintech	15613-1-AP
	Collagen Type I Monoclonal	Proteintech	66761-1-Ig
	Phospho-Jak2 (Tyr1007/1008)	Cell Signaling Technology	3771S
	Jak2	Cell Signaling Technology	3230
Secondary antibodies	HRP was used to label goat anti-mouse IgG	Cell Signaling Technology	7076S
	HRP was used to label goat anti-rabbit IgG	Cell Signaling Technology	7074S
	CoraLite 488 - conjugated Affinipure Goat Anti-Rabbit IgG (H+L)	Proteintech	SA00013-2
