# Supplementary material for: DST-3, a Novel Modified Cryptotanshinone, Protects Against Pulmonary Fibrosis via Inhibiting STAT3/Smad Signaling Pathway and Improves Bioavailability
Source: Pharmaceutics. 2025 Oct 8;17(10):1307. doi: 10.3390/pharmaceutics17101307 (PMC12566936; doi:10.3390/pharmaceutics17101307)
Supplement: Supplementary file 1 [file pharmaceutics-17-01307-s001.zip › Supplement Material S3 - Validation of methodology.pdf]

1. Methodological confirmation

According to the guidelines, comprehensive method confirmation should be carried out for the first time established biological sample analysis methods, new drugs or new metabolites quantitative analysis. In this study, the verification of DST-3 analysis methods in biological samples from SD rats was comprehensive confirmation, including specificity, linear range, precision, accuracy, matrix effect and recovery rate.

1.1. Specificity

Blank samples, low concentration DST-3 quality control samples and biological samples from 6 different rats after intragastric administration of 60 mg/kg DST-3 were taken, respectively. After treatment, samples were injected and analyzed to obtain chromatograms of DST-3 and LTD, and to analyze whether the endogenous substances in the biological samples interfered with DST-3 and LTD.

1.2. Linear Range

DST-3 standard curve samples were taken and processed for HPLC-MS/MS injection analysis to obtain corresponding chromatograms. The drug concentration of DST-3 (ng/mL) was taken as the horizontal coordinate and the ratio of chromatographic peak area between DST-3 and internal standard LTD was taken as the vertical coordinate for linear regression analysis. According to the sample concentration of DST-3 standard curve, the linear range of the analysis method was determined, and the phase relation value (R^2) was tested to see whether

it met the requirements.

Table S3-1. Calibration curve parameters for DST-3 and internal standard (LTD)

Compound	Calibration range (ng/mL)	LLOQ (ng/mL)	Retention time (min)	R^2
DST-3	2–500	2	4.15	>0.99
LTD (IS)	–	–	1.76	–

1.3. Lower Limit of Quantitation (LLOQ)

The lower limit of quantification refers to the lowest concentration that can be accurately quantified by the instrument. Six DST-3 biological samples with a concentration of 2 ng/mL were prepared and analyzed by HPLC-MS/MS after treatment. The peak area ratio between DST-3 and LTD was calculated. The peak area ratio was substituted into the standard curve, and the concentration of 6 samples was calculated. Compared with the standard concentration, the precision was reflected by RSD, which should be less than 20%, the accuracy should be within the range of 80-120%, and the accuracy was reflected by RE, which should be within the range of -20%-20%.

1.4. Precision and accuracy

DST-3 quality control samples (5 samples in low, medium and high concentration) were taken, and accompanying standard curves were prepared. After treatment, HPLC-MS/MS sampling analysis was performed. Calculate the peak area ratio between DST-3 and LTD, substitute the peak area ratio into the standard curve, calculate the concentration of the quality control sample, and compare with the standard concentration to calculate the precision and accuracy in the batch. According to the requirements of the guidelines, the precision should be

reflected by RSD. The RE should be in the range of -20%-20%. The same method was used for continuous determination for 3 days to calculate the precision and accuracy between batches. The precision RSD were also required to be less than 20%, and the accuracy RE was within the range of -20%-20%.

Table S3-2. Precision and accuracy of DST-3 in rat plasma and tissues (n = 6)

**Sample Type**	**QC Level**	**Nominal Conc. (ng/mL)**	**RE (%)**	**RSD (%)**	**Criteria**
Plasma	LLOQ	2	10.0	9.0	Within ±20%, ≤20%
Plasma	Low	–	–2.8 ~ 5.6	–1.0 ~ 8.6	Within ±20%, ≤20%
Plasma	Medium	–	–1.0 ~ –6.7	4.6 ~ 14.4	Within ±20%, ≤20%
Plasma	High	–	–5.4 ~ 4.5	6.1 ~ 14.1	Within ±20%, ≤20%
Tissue	Low	–	–1.2 ~ 8.9	4.9 ~ 11.4	Within ±20%, ≤20%
Tissue	Medium	–	–4.7 ~ –0.8	7.9 ~ 14.5	Within ±20%, ≤20%
Tissue	High	–	–0.7 ~ 2.8	6.9 ~ 11.1	Within ±20%, ≤20%

1.5. Sample Stability

DST-3 quality control samples (5 samples in low and high concentration) were taken, and accompanying standard curves were prepared. The samples were stored at normal temperature for 48 h or -80 °C for 1 month, and then analyzed by HPLC-MS/MS. The peak area ratio between DST-3 and LTD was calculated. The peak area ratio was substituted into the standard curve to calculate the sample concentration. Compared with the standard concentration, according to the guidelines, RE should be in the range of -20%-20% for both low and high concentration results, and RSD should be less than 20%.

1.6. Matrix effect

DST-3 quality control samples (12 samples in low and high concentration) were taken, and

accompanying standard curves were prepared. After treatment, HPLC-MS/MS samples were injected immediately for analysis. For each batch of substrate, the matrix factor for each analyte and the inner target should be calculated by calculating the ratio of the peak area in the presence of the substrate (measured by extracting the blank substrate and adding the analyte and inner target) to the corresponding peak area (analyte and inner target pure solution) without the substrate. Furthermore, the matrix factors normalized by internal standard were calculated by dividing the matrix factors of the analyte into the underlying matrix factors. The coefficient of variation of the internal standard normalized matrix factors calculated from 6 batches of matrix should not be bigger than 15%. Detailed parameters were provided in Table S3-3.

Table S3-3. Stability and matrix effect of DST-3 in rat plasma and tissues

**Condition**	**QC Level**	**RE (%)**	**RSD (%)**	**Criteria**
Room temp (48 h)	Low	–19.2 ~ 3.5	7.3 ~ 13.8	Within ±20%, ≤20%
–80 °C (1 month)	Low	–12.4 ~ 8.5	5.0 ~ 16.9	Within ±20%, ≤20%
Room temp (48 h)	High	–9.8 ~ 10.5	2.7 ~ 11.0	Within ±20%, ≤20%
–80 °C (1 month)	High	–8.2 ~ 8.2	4.5 ~ 18.5	Within ±20%, ≤20%
Matrix effect (tissue/plasma)	Low	–3.6 ~ 0.1	–3.4 ~ 4.1	Acceptable if ≈1

1.7. Recovery rate

DST-3 quality control samples (12 samples in low, medium and high concentration) were taken, among which 3 samples of low, medium and high concentration were treated with biological samples and the other 3 samples were not treated. Finally, HPLC-MS/MS samples were immediately injected for analysis. Calculate the peak area ratio of DST-3, compare with the standard concentration, calculate the recovery rate, and the results should be precise.

2. Experimental Results

2.1. Specificity

Using the optimized HPLC-MS/MS analysis method, blank plasma and tissue samples, low concentration DST-3 quality control samples, and biological samples after 60 mg/kg DST-3 intragastric administration were detected, and specific chromatograms of DST-3 and internal standard LTD were obtained, as shown in Figure S3-1. The peak time of DST-3 and Internal standard LTD is about 4.15 min and 1.76 min respectively. There is no interference between each other, and no interference from internal substances, indicating that the proposed analytical method is highly specific.

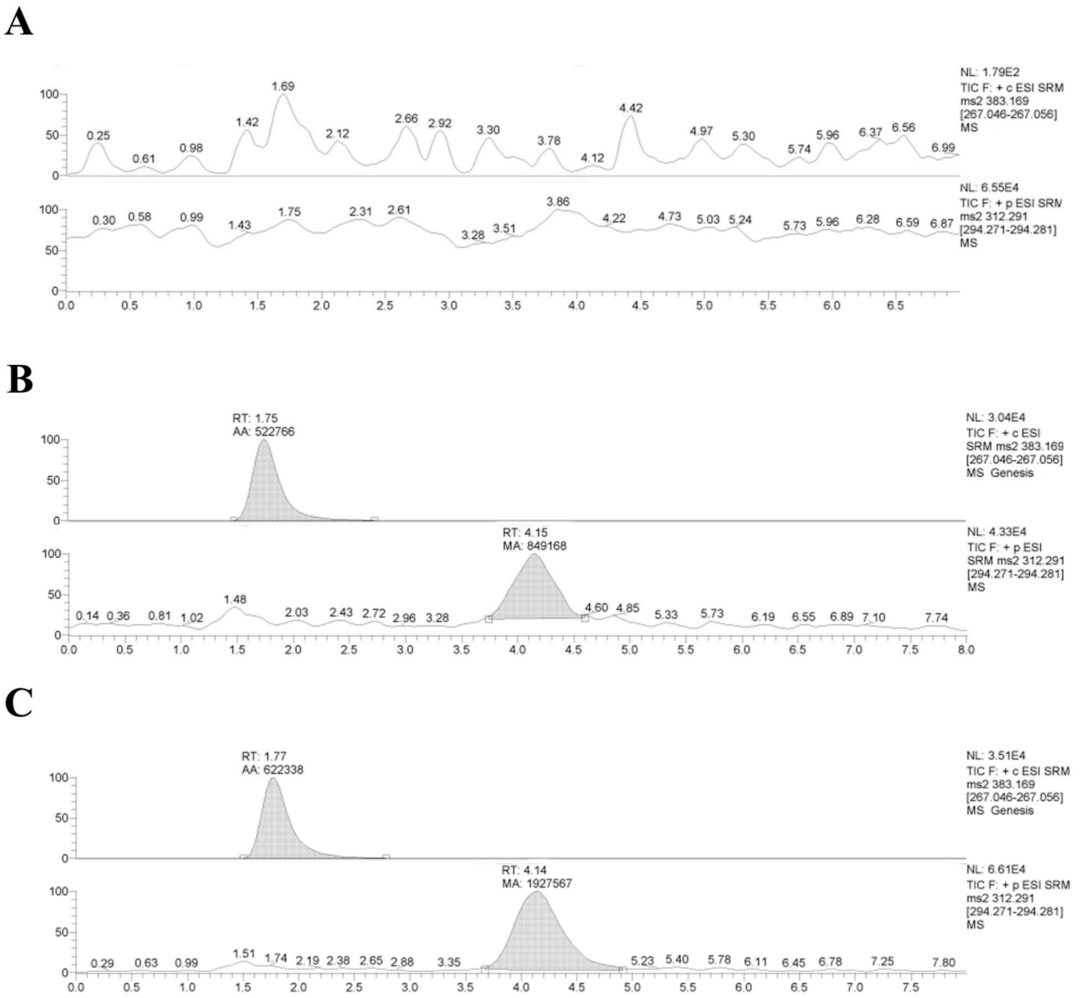

Figure S3-1. The Chromatograms of DST-3 and LTD in rat plasma. A: Samples of rat blank plasma; B: Low concentration quality control samples of DST-3; C: Samples of rat plasma collected at 15 min after intragastric administration of DST-3;

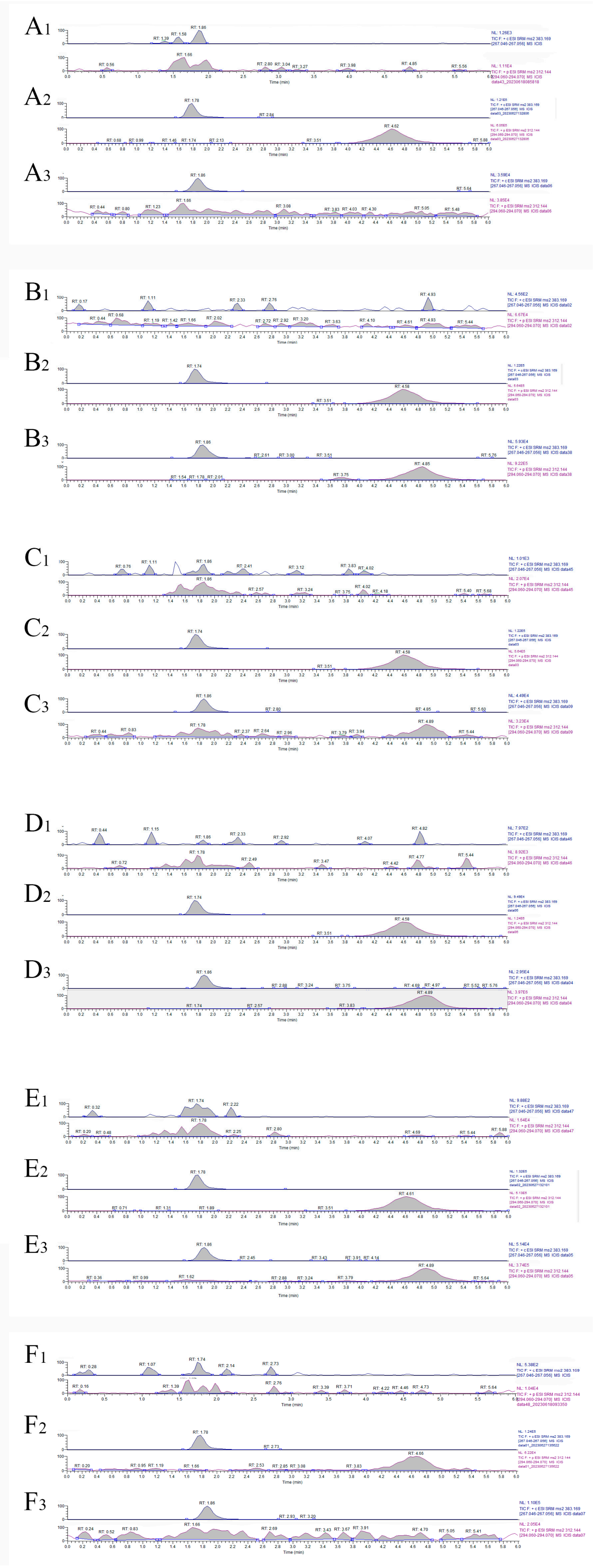
A1
A2
A3
B1
B2
B3
C1
C2
C3
D1
D2
D3
E1
E2
E3
F1
F2
F3

Figure S3-2. The Chromatograms of DST-3 and LTD in rat tissue. A: heart; B: liver; C: spleen; D: lung; E: kidney; F: brain; (1: Samples of rat blank plasma; 2: Low concentration quality control samples of DST-3; 3: Samples of rat tissue collected at 15 min after intragastric administration of DST-3)

2.2. Linear Range

According to the method under "1.2 Linear Range", the standard curve of plasma was obtained with the concentration of DST-3 as the horizontal coordinate and the ratio of peak area of DST-3 to peak area of internal standard LTD as the vertical coordinate. The linear range of plasma was 2-200 ng/mL. According to the results of linear regression analysis, the regression equation and correlation coefficient of standard curve were obtained. The regression equation of standard curve accompanying multiple analysis batches of plasma samples was shown in Table S3-1. The results showed that the ratio of DST-3 peak area to internal standard LTD peak area had a linear relationship with DST-3 concentration in the linear range, and the correlation coefficient was good. The linear range of DST-3 concentration in plasma samples was 2-200 ng/mL.

Table S3-4. Regression equation of DST-3 standard curve in rat plasma

Compound	Type	Regression equation	Correlation coefficient (R^2)	Liner range (ng /mL)
DST-3	Blood	Y=1.6775+0.0819*X	0.9983	2-500
	Heart	Y=0.9027+0.0458*X	0.9968	2-500

	Liver	Y=1.0518+0.058*X	0.9942	2-500
	Spleen	Y=0.362+0.0431*X	0.9993	2-500
	Lung	Y=0.1575+0.0501*X	0.9986	2-500
	Kidney	Y=-0.2644+0.0645*X	0.9958	2-500
	Brain	Y=-0.0052+0.556*X	0.9970	2-500
	Blood	Y=-0.6076+0.3626*X	0.9981	2-500
	Heart	Y=1.6297+0.1744*X	0.9915	2-500
	Liver	Y=5.7277+0.2152*X	0.9990	2-500
CTS	Spleen	Y=4.0962+0.1891*X	0.9928	2-500
	Lung	Y=5.0222+0.2067*X	0.9971	2-500
	Kidney	Y=4.9334+0.2423*X	0.9952	2-500
	Brain	Y=0.3931+0.1149*X	0.9995	2-500

2.3. Lower limit of Quantification

As for the HPLC-MS/MS analysis method established in this study, the lower limit of quantification of DST-3 in plasma and tissue samples was 2 ng/mL, and the confirmed results of lower limit of quantification were shown in Table S3-2. The results showed that the RE or RSD of these LLOQ samples in heart, liver, spleen, lung, kidney, brain and plasma were 10.0%、-2.8%, 1.9%, 0.8%, 0.4%, -1.6% and 5.6% or 9.0%, 8.3%, 15.2%, 16.4%, 11.8%, 13.9% and 11.5%, which met the requirements of the guidelines.

Table S3-5. LLOQ of DST-3 in rat plasma samples

Number		Plasma Concentration (ng/mL)		
		2.0	20.0	200.0
Measured concentration (ng/mL)	1	2.1	21.4	190.1
	2	2.4	22.3	196.0
	3	2.0	23.5	185.1
	4	2.4	18.3	189.1
	5	2.3	20.7	198.5
	6	1.9	19.5	189.1
Mean (ng/mL)		2.2	18.8	191.3
SD		0.2	1.0	5.0
RSD (%)		10.0	5.2	2.6
RE (%)		9.0	-6.2	-4.5

Table S3-6. LLOQ of DST-3 in rat tissue samples

Tissue	Concentration (ng/mL)	Measured con. (ng/mL)		*RSD* (%)	*RE* (%)
		Mean	***SD***		
Heart	2	1.9	0.2	8.3	-2.8
Liver	2	2.0	0.3	15.2	1.9
Spleen	2	2.0	0.3	16.4	0.8
Lung	2	2.0	0.2	11.8	0.4

Kidney	2	2.0	0.3	13.9	-1.6
Brain	2	2.1	0.2	11.5	5.6

2.4. Precision accuracy in batch

The plasma, heart, liver, spleen, lung, kidney and brain quality control samples of the same batch were analyzed, and the RE or RSD of low-concentration quality control samples were -0.0%, -2.8%, 1.9%, 0.8%, 0.4%, -1.6% and 5.6% or 8.6%, 8.3%, 15.2%, 16.4%, 11.8%, 13.9% and 11.5%; Medium concentration in the quality control samples of RE or RSD were -6.7%, 1.0%, 1.7%, 1.4%, 2.3%, 0.3% and 5.9% or 10.5%, 7.8%, 4.6%, 14.8%, 9.0%, 14.4% and 12.6%; High concentration of quality control samples of RE or RSD were -5.4%, 0.6%, 1.4%, 4.2%, 4.5%, 2.9% and 2.4% or 10.1%, 6.1%, 14.1%, 10.2%, 12.5%, 6.4% and 9.3%; For each quality control sample of the same batch, RE of low, medium and high concentration results were all in the range of -20%-20%, and RSD were all less than 20%. The results are shown in Table S3-7 and S3-8. The results were in line with the guidelines and met the analysis requirements.

Table S3-7. Intra-batch precision and accuracy of DST-3 in rat plasma samples

**Number**	**Concentration (ng/mL)**		
	**2.0**	**20.0**	**200.0**
1	2.0	17.5	205.5
2	1.9	16.7	194.9
3	2.0	21.8	223.3

4	2.2	19.7	182.7
5	1.7	17.1	220.2
6	2.1	19.5	241.7
Mean	2.0	18.7	211.4
SD	0.2	2.0	21.3
RSD (%)	8.6	10.5	10.1
RE (%)	-0.0	-6.7	5.4

Table S3-8. Intra-batch precision and accuracy of DST-3 in rat tissue samples

**Tissue**	**Concentration (ng/mL)**	**Measured con. (ng/mL)**		***RSD* (%)**	***RE* (%)**
		Mean	***SD***		
Heart	2	1.9	0.2	8.3	-2.8
	20	19.8	1.6	7.8	-1.0
	200	201.3	12.2	6.1	0.6
Liver	2	2.0	0.3	15.2	1.9
	20	20.3	0.9	4.6	1.7
	200	202.8	28.6	14.1	1.4
Spleen	2	2.0	0.3	16.4	0.8
	20	20.3	3.0	14.8	1.4
	200	208.7	21.2	10.2	4.2

Lung	2	2.0	0.2	11.8	0.4
	20	20.5	1.8	9.0	2.3
	200	209.3	26.1	12.5	4.5
Kidney	2	2.0	0.3	13.9	-1.6
	20	19.9	2.9	14.4	-0.3
	200	194.4	12.5	6.4	-2.9
Brain	2	2.1	0.2	11.5	5.6
	20	21.3	2.7	12.6	5.9
	200	205.0	19.0	9.3	2.4

2.5. Precision accuracy between batches

The plasma, heart, liver, spleen, lung, kidney and brain quality control samples of the three consecutive days were analyzed, and the RE or RSD of low-concentration quality control samples were 8.9 %、-1.2%、0.6%、1.7%、2.3%、-0.6% and 2.5% or 4.9 %、9.5%、10.4%、13.4%、8.6%、11.1% and 11.4%; Medium concentration in the quality control samples of RE or RSD were -4.7%、0.6%、0.4%、1.8%、2.9%、-0.8% and 3.4% or -4.7%、0.6%、0.4%、1.8%、2.9%、-0.8% and 3.4%; High concentration of quality control samples of RE or RSD were -1.4%、0.9%、-0.7%、1.4%、2.8%、0.1% and 1.8% or 8.5%、6.9%、11.1%、8.8%、10.5%、8.4% and 10.9%; For each quality control sample of the same batch, RE of low, medium and high concentration results were all in the range of -20%-20%, and RSD were all less than 20%. The results are shown in Table S3-9 and S3-10. The results were in line with the guidelines

and met the analysis requirements.

Table S3-9. Inter-batch precision and accuracy of DST-3 in rat plasma samples

Batch number	Number	Concentration (ng/mL)		
		**2.0**	**20.0**	**200.0**
1	1	2.0	17.5	205.5
	2	1.9	16.7	194.9
	3	2.0	21.8	223.3
	4	2.2	19.7	182.7
	5	1.7	17.1	220.2
	6	2.1	19.5	241.7
2	1	2.1	21.4	190.1
	2	2.4	22.3	196.0
	3	2.0	23.5	185.1
	4	2.4	18.3	189.1
	5	2.3	20.7	198.5
	6	1.8	19.5	189.1
3	1	2.1	18.0	186.3
	2	2.3	18.0	187.3
	3	2.0	15.9	184.3
	4	2.0	18.4	208.8

	5	2.2	18.4	178.1
	6	2.1	17.1	189.2
Mean		2.1	18.8	197.2
SD		0.2	2.1	16.7
RSD (%)		8.9	11.1	8.5
RE (%)		4.9	-4.7	-1.4

Table S3-10. Inter-batch precision and accuracy of DST-3 in rat tissue samples

**Tissue**	**Concentration(ng/mL)**	**Measured con. (ng/mL)**		***RSD* (%)**	***RE* (%)**
		Mean	***SD***		
Heart	2	2.0	0.2	9.5	-1.2
	20	20.1	1.6	7.9	0.6
	200	201.7	14.0	6.9	0.9
Liver	2	2.0	0.2	10.4	0.6
	20	20.1	2.1	10.3	0.4
	200	198.6	22.1	11.1	-0.7
Spleen	2	2.0	0.3	13.4	1.7
	20	20.4	2.5	12.1	1.8
	200	202.8	17.9	8.8	1.4
Lung	2	2.0	0.2	8.6	2.3

	20	20.6	2.1	10.0	2.9
	200	205.8	21.6	10.5	2.8
	2	2.0	0.2	11.1	-0.6
Kidney	20	19.8	2.2	11.2	-0.8
	200	200.2	16.7	8.4	0.1
	2	2.0	0.2	11.4	2.5
Brain	20	20.7	3.0	14.5	3.4
	200	203.7	22.1	10.9	1.8

2.6. Stability

The same batch of plasma, heart, liver, spleen, lung, kidney and brain tissue frozen at room temperature for 48h or -80°C for 1 month with low and high concentration quality control samples were analyzed. The RE of the low concentration plasma quality control samples were 3.5%, 19.2%, 7.1%, 14.1%, 5.2%, 5.2%, 7.2% or 8.5%, to 12.4%, 11.4%, 4.6%, 10.3%, 9.7%, 11.4%, RSD were 13.8%, 12.5%, 11.9%, 11.1%, 10.9%, 9.7%, 7.3%, or 16.9%, 9.2%, 15.4%, 10.0%, 8.3%, 9.3%, 5.0%; The RE of the high concentration plasma quality control samples were 10.5%, 5.2%, 9.8%, 1.4%, 6.2%, 7.9%, 1.4% or 8.2%, 3.7%, 4.6%, 4.7%, 8.2%, 4.3%, 6.4%, RSD were 11.0%, 8.1%, 7.8%, 6.7%, 3.8%, 5.9%, 2.7% or 18.5%, 6.2%, 8.6%, 4.5%, 8.0%, 9.1%, 9.5%. RE in both low and high concentration results was in the range of -20%-20%, and RSD was less than 20%, which complied with the guidelines. The results are shown in Table S3-11 and S3-12.

Table S3-11. Stability of DST-3 in rat plasma samples

Number	Concentration (48 h, ng/mL)		Concentration (1 M, ng/mL)	
	**2.0**	**200.0**	**2.0**	**200.0**
1	2.4	198.2	2.3	227.3
2	1.6	229.3	1.8	212.3
3	2.3	251.0	1.6	154.4
4	2.1	191.0	1.5	190.0
5	1.8	212.9	2.1	137.2
6	2.1	243.2	1.8	180.4
Mean	2.1	220.9	1.8	183.6
SD	0.3	24.3	0.3	34.0
RSD (%)	13.8	11.0	16.9	18.5
RE (%)	3.5	10.5	-8.5	-8.2

Table S3-12. Stability of DST-3 in rat tissue samples

Tissue	Time	Concentration (ng/mL)	Measured con. (ng/mL)		***RSD* (%)**	***RE* (%)**
			Mean	***SD***		
Heart	48 h	2	1.7	0.2	12.5	-19.2
		200	190.1	15.4	8.1	-5.2
	1 M	2	1.8	0.2	9.2	-12.4

		200	192.9	11.9	6.2	-3.7
Liver	48 h	2	1.9	0.2	11.9	-7.1
		200	182.1	14.3	7.8	-9.8
	1M	2	1.8	0.3	15.4	-11.4
		200	191.3	16.4	8.6	-4.6
Spleen	48 h	2	1.8	0.2	11.1	-14.1
		200	197.3	16.3	6.7	-1.4
	1 M	2	1.9	0.2	10.0	-4.6
		200	1.8	0.2	8.3	-10.3
Lung	48 h	2	1.9	0.2	10.9	-5.2
		200	188.3	7.2	3.8	-6.2
	1 M	2	191.0	8.5	4.5	-4.7
		200	184.9	14.8	8.0	-8.2
Kidney	48 h	2	1.9	0.2	9.7	-5.2
		200	185.4	11.0	5.9	-7.9
	1 M	2	1.8	0.2	9.3	-9.7
		200	191.8	17.5	9.1	-4.3
Brain	48 h	2	1.9	0.1	7.3	-7.2
		200	197.2	5.2	2.7	-1.4
	1 M	2	1.8	0.1	5.0	-11.4

	200	187.9	17.9	9.5	-6.4

2.7. Matrix effect

The low-concentration quality control samples of plasma, heart, liver, spleen, lung, kidney and brain from the same batch were analyzed. The average ratio of low-concentration DST-3 standard solution to low-concentration quality control samples of each group was 1.00, 0.97, 1.02, 0.98, 0.96, 0.99 and 0.97. RSD were 4.1%, 3.3%, 4.8%, 6.9%, 7.7%, 7.7% and 9.7%, RE were 0.1%, 3.4%, 1.7%, 1.9%, 3.6%, 0.6% and 2.7%. The matrix effect of the same batch of low-concentration quality control samples was low, and RSD were all less than 10%, as shown in Table S3-13 and 3-14. The results were in line with the guidelines and met the analysis requirements.

Table S3-13. Effect of blank plasma on DST-3 matrix in rats

**Number**	**Compound (pure solution / blank plasma)**	
	**DST-3**	**LTD**
1	0.86	0.88
2	0.80	0.79
3	0.77	0.70
4	0.87	0.87
5	0.81	0.80
6	0.69	0.69
Mean	1.00	

SD	0.04
RSD (%)	4.1
RE (%)	0.1

Table S3-14. Effect of tissue on DST-3 matrix in rats

**Tissue**	**Concentration (ng/mL)**	**Measured con. (ng/mL)**		***RSD* (%)**	***RE* (%)**
		Mean	***SD***		
Heart	2	0.96	0.03	3.3	-3.4
Liver	2	1.02	0.05	4.8	-1.7
Spleen	2	0.98	0.07	6.9	-1.9
Lung	2	0.96	0.07	7.7	-3.6
Kidney	2	0.99	0.08	7.7	-0.6
Brain	2	0.97	0.09	9.7	-2.7

2.8. Recovery rate

The low, medium and high concentration quality control samples of plasma, heart, liver, spleen, lung, kidney and brain tissue from the same batch were analyzed. The Recovery rate was calculated as: Recovery (%) = (Measured concentration of processed sample / Measured concentration of unprocessed sample) × 100%. The recovery rate of all concentration quality control samples in the same batch should be within 50-110%, and RSD should be less than 20%, as shown in Table S3-15 and S3-16. The results are in line with the guidelines and meet the

analysis requirements.

Table S3-15. Determination of recovery rate accuracy of DST-3 in plasma samples

**Concentration (ng/mL)**	**Measured con. (%)**		***RSD*** **(%)**	***Criteria***
	Mean	***SD***		
2	98.2	6.9	7.2	RSD ≤ 20%
20	76.4	8.2	10.5	Recover rate
200	51.3	8.8	8.1	within 50-110%

Table S3-16. Determination of recovery rate accuracy of DST-3 in tissue samples

**Tissue**	**Concentration (ng/mL)**	**Measured con. (%)**		***RSD*** **(%)**	***Criteria***
		Mean	***SD***		
	2	92.8	9.5	9.0	RSD ≤ 20%
Heart	20	74.5	8.3	8.9	Recover rate
	200	50.9	9.8	12.8	within 50-110%
	2	101.9	10.2	10.3	RSD ≤ 20%
Liver	20	89.1	9.9	11.1	Recover rate
	200	62.5	10.5	14.5	within 50-110%
	2	76.1	12.1	11.9	RSD ≤ 20%
Spleen	20	65.8	10.8	12.2	Recover rate
	200	53.8	9.5	13.3	within 50-110%

	2	54.8	11.3	11.8	RSD ≤ 20%
Lung	20	68.0	12.5	14.4	Recover rate
	200	49.4	10.2	18.8	within 50-110%
	2	73.4	8.9	8.6	RSD ≤ 20%
Kidney	20	72.3	10.1	10.7	Recover rate
	200	51.8	9.7	12.2	within 50-110%
	2	52.3	11.8	12.3	RSD ≤ 20%
Brain	20	66.1	12.2	14.3	Recover rate
	200	54.3	10.5	15.0	within 50-110%

2.9. Pharmacokinetics of DST-3 in SD rats after single intravenous or oral injection

After a single intravenous injection of 6 mg/kg or oral administration of 60 mg/kg DST-3 or CTS in SD rats, plasma DST-3 concentration data were measured at different time points, as shown in Table S3-17 and S3-18. Plasma CTS concentration data were measured at different time points, as shown in Table S3-17, S3-18, S3-19 and S3-20.

Table S3-17. Plasma concentration of DST-3 in SD rats after a single intravenous injection of 6 mg/kg (ng/mL)

**Time (h)**	**Number**				***Mean***	***SD***
	**1**	**2**	**3**	**4**		
0	ND	ND	ND	ND	ND	ND
0.25	391.5	343.0	319.9	312.1	341.6	31.0

0.5	195.2	250.0	175.5	173.5	198.6	30.9
1	78.1	170.6	71.5	134.0	113.5	40.9
2	53.8	82.1	93.1	41.9	67.7	20.7
3	65.9	40.5	68.2	27.7	50.6	17.1
4	37.6	12.0	27.1	28.0	26.1	9.2
6	24.0	17.4	20.0	23.9	21.3	2.8
8	14.3	2.0	12.2	8.6	9.3	4.6
12	5.2	ND	10.4	10.9	8.8	2.6
24	6.4	5.0	1.6	2.6	3.9	1.9

TableS3-18. Plasma concentration of DST-3 in SD rats after single oral administration of 60 mg/kg (ng/mL)

**Time (h)**	**Number**				***Mean***	***SD***
	**5**	**6**	**7**	**8**		
0	ND	ND	ND	ND	ND	ND
0.25	16.9	1.5	1.5	1.7	5.4	6.6
0.5	15.2	16.5	8.1	7.4	11.8	4.1
1	25.1	45.6	80.3	27.4	44.6	22.1
2	63.6	89.7	92.0	61.6	76.7	14.2
3	97.9	95.9	143.4	122.5	114.9	19.5

4	67.0	79.7	131.5	66.6	86.2	26.7
6	52.2	46.4	123.0	59.1	70.2	30.8
8	37.2	36.2	100.2	31.5	51.2	28.3
12	21.8	20.9	57.3	21.5	30.4	15.5
24	9.0	7.8	3.4	ND	6.7	2.4

Table S3-19. Plasma concentration of CTS in SD rats after a single intravenous injection of 6 mg/kg (ng/mL)

Time (h)	Number				*Mean*	*SD*
	1	2	3	4		
0	ND	ND	ND	ND	ND	ND
0.25	360.9	347.3	337.1	320.0	341.3	14.9
0.5	166.6	187.7	159.3	153.0	166.6	13.1
1	51.7	131.5	87.5	96.0	91.7	28.3
2	52.5	110.1	56.9	76.2	73.9	22.7
3	52.2	79.7	55.0	64.5	62.8	10.7
4	28.2	63.5	38.4	52.8	45.7	13.5
6	21.4	49.9	16.4	32.0	29.9	12.8
8	16.4	30.0	12.1	17.5	19.0	6.7
12	10.1	11.7	8.6	10.2	10.2	1.1

24	4.5	6.8	4.7	3.3	4.8	1.3

Table S3-20. Plasma concentration of CTS in SD rats after single oral administration of 60 mg/kg (ng/mL)

Time (h)	Number				*Mean*	*SD*
	**5**	**6**	**7**	**8**		
0	ND	ND	ND	ND	ND	ND
0.25	15.7	10.9	13.5	6.6	11.7	3.4
0.5	16.7	8.1	11.9	17.7	13.6	3.9
1	17.3	11.3	20.1	27.0	18.9	5.6
2	15.0	13.2	14.2	23.0	16.3	3.9
3	25.4	27.3	19.6	19.5	23.0	3.4
4	42.8	44.6	22.3	29.6	34.8	9.3
6	22.9	30.2	41.1	23.2	29.4	7.4
8	33.9	32.4	31.8	25.7	31.0	3.1
12	17.8	15.5	20.2	16.5	17.5	1.8
24	8.1	11.4	7.4	8.7	8.9	1.5
